# Combining Metabolite-Based Pharmacophores with Bayesian Machine Learning Models for *Mycobacterium tuberculosis* Drug Discovery

**DOI:** 10.1371/journal.pone.0141076

**Published:** 2015-10-30

**Authors:** Sean Ekins, Peter B. Madrid, Malabika Sarker, Shao-Gang Li, Nisha Mittal, Pradeep Kumar, Xin Wang, Thomas P. Stratton, Matthew Zimmerman, Carolyn Talcott, Pauline Bourbon, Mike Travers, Maneesh Yadav, Joel S. Freundlich

**Affiliations:** 1 Collaborative Drug Discovery Inc., 1633 Bayshore Highway, Suite 342, Burlingame, CA, 94010, United States of America; 2 Collaborations in Chemistry, 5616 Hilltop Needmore Road, Fuquay-Varina, NC, 27526, United States of America; 3 SRI International, 333 Ravenswood Avenue, Menlo Park, CA, 94025, United States of America; 4 Departments of Pharmacology & Physiology and Medicine, Center for Emerging and Reemerging Pathogens, Rutgers University–New Jersey Medical School, 185 South Orange Avenue, Newark, NJ, 07103, United States of America; 5 Department of Medicine, Center for Emerging and Reemerging Pathogens, Rutgers University–New Jersey Medical School, 185 South Orange Avenue, Newark, NJ, 07103, United States of America; 6 Public Health Research Institute, Rutgers University–New Jersey Medical School, Newark, NJ, 07103, United States of America; University of Padova, Medical School, ITALY

## Abstract

Integrated computational approaches for *Mycobacterium tuberculosis* (*Mtb*) are useful to identify new molecules that could lead to future tuberculosis (TB) drugs. Our approach uses information derived from the TBCyc pathway and genome database, the Collaborative Drug Discovery TB database combined with 3D pharmacophores and dual event Bayesian models of whole-cell activity and lack of cytotoxicity. We have prioritized a large number of molecules that may act as mimics of substrates and metabolites in the TB metabolome. We computationally searched over 200,000 commercial molecules using 66 pharmacophores based on substrates and metabolites from *Mtb* and further filtering with Bayesian models. We ultimately tested 110 compounds *in vitro* that resulted in two compounds of interest, BAS 04912643 and BAS 00623753 (MIC of 2.5 and 5 μg/mL, respectively). These molecules were used as a starting point for hit-to-lead optimization. The most promising class proved to be the quinoxaline di*-N*-oxides, evidenced by transcriptional profiling to induce mRNA level perturbations most closely resembling known protonophores. One of these, SRI58 exhibited an MIC = 1.25 μg/mL versus *Mtb* and a CC_50_ in Vero cells of >40 μg/mL, while featuring fair Caco-2 A-B permeability (2.3 x 10^−6^ cm/s), kinetic solubility (125 μM at pH 7.4 in PBS) and mouse metabolic stability (63.6% remaining after 1 h incubation with mouse liver microsomes). Despite demonstration of how a combined bioinformatics/cheminformatics approach afforded a small molecule with promising *in vitro* profiles, we found that SRI58 did not exhibit quantifiable blood levels in mice.

## Introduction

Learning from experience in neglected disease drug discovery is essential for increasing time- and cost efficiencies. This requires we leverage and build upon computational methods widely used in industrial drug discovery [1, 2]. For example, we have previously analyzed large datasets for *Mycobacterium tuberculosis* (*Mtb*) [3–14]–the causative agent of tuberculosis (TB). We have used these to build machine learning models that use single point data, dose response data [3, 4], combine bioactivity and cytotoxicity data (e.g, Vero, HepG2 or other cells) [8–10] or combinations of the preceding [13, 15]. The deliverables have been promising novel (or long-abandoned) antitubercular hits for further pursuit as well as strategies for hit-to-lead evolution and prediction of antitubercular *in vivo* activity in the mouse model of infection [14].

Whole-cell phenotypic high-throughput screening (HTS) against *Mtb* does not typically provide information on the potential target/s for the compounds and so other methods must be used for target identification [16, 17]. For example, we have contributed computational methods that rely on similarity of compounds to inhibitors of known targets [17] to create TB Mobile 2 which applies a machine learning approach to predict target likelihood.

Since a small fraction of *Mtb* proteins are known to be modulated by approved TB drugs [7], a need exists to modulate other targets to avoid existing drug resistance mechanisms. We have focused initially on the targets that were essential to the growth and survival of *Mtb* [18], under *in vitro* and *in vivo* conditions [19], and ultimately declared respective lists of essential enzymes and their essential metabolites [6, 7]. In an effort to discover inhibitors of 9 essential enzymes through their mimicry of the chemical structure of a given metabolite, 3D pharmacophores were used to screen over 80,000 commercial compounds. Ultimately after testing 23 candidate inhibitors or metabolite mimics (including 3 predicted inactives), 2 moderately active compounds were identified [7]. In the current study we have greatly expanded our approach to also assess targets that are *in vitro* but not *in vivo* essential. We computationally searched >206,000 molecules with 66 pharmacophores of *Mtb* essential metabolites or substrates and assayed 110 compounds *in vitro*. We have identified 3 compounds possessing whole-cell activity against *Mtb*. Two of the hits were further optimized in a drug discovery workflow. We demonstrate that this approach of computational metabolite mimicry is scalable to afford promising chemical entities and could be explored for other diseases and yet it is ultimately confounded by molecular properties that impact *in vivo* pharmacokinetics.

## Results

### Small molecule information from CDD for new potential *Mtb* enzyme targets

Except for one of the 46 potential targets (MurE) identified (S1 Table) in our initial bioinformatics analysis (See Materials and Methods), none of the enzymes described have any small molecule inhibitors noted in the CDD Public database at the time of this study. Depending on various criteria like *in vivo* essentiality, whether or not X-ray crystallographic information was available in the Protein Data Bank (www.rcsb.org), a subjective interest in the constituent pathway/s, suitability of the structure for the enzyme substrate or product to facilitate mimic design (e.g. lack of charge), 20 targets were selected from the list of 46 enzymes as being of particular interest. These are TrpB, MetE, IlvD, FolK, HisC1, HsaE, **End, BioF1, CobL, Ace, AccD1, SerB2, AmiD, HsaF, Rv1879, Tal, FabG, NuoD, ProA, and ArcA** (bold are those encoded by a gene predicted to be *in vivo* essential, (S2 Table)). Reaction details including substrates and products (and their relevant SMILES strings) are provided for these 20 selected targets. As described previously [7] the TBcyc pathway database (http://tbcyc.tbdb.org/index.shtml), an *Mtb* specific metabolic pathway database, was used to extract this information (S2 Table). The TBcyc database was initially developed using SRI's Pathway Tools software that automatically generates a Pathway/Genome Database (PGDB) describing the genome and biochemical networks of the organism from the annotated genome sequence of *Mtb* [20, 21].

### *In silico* selection of putative metabolite and substrate mimics

14,733 commercial molecules were retrieved from over a set of 206,000 (from the Asinex Gold library) using the 66 pharmacophores (S1 Fig and S1 Model Files) based on enzymatic reaction substrate and product chemical structures and were suggested as potential mimics. These molecules were scored with three dual event Bayesian *Mtb* models (MLSMR, CB2, Kinase) [10, 22–25] in Discovery Studio [4, 26, 27]. All compounds were imported into CDD. 110 molecules were selected for purchase given pharmacophore scores greater than 2.5 (higher scores are better), ‘active’ scores in all 3 dual event models, and successful visual filtering (e.g., absence of reactive functional groups) [28].

### Measurement of Antibacterial Activity Against *Mtb*

**Fig 1:**
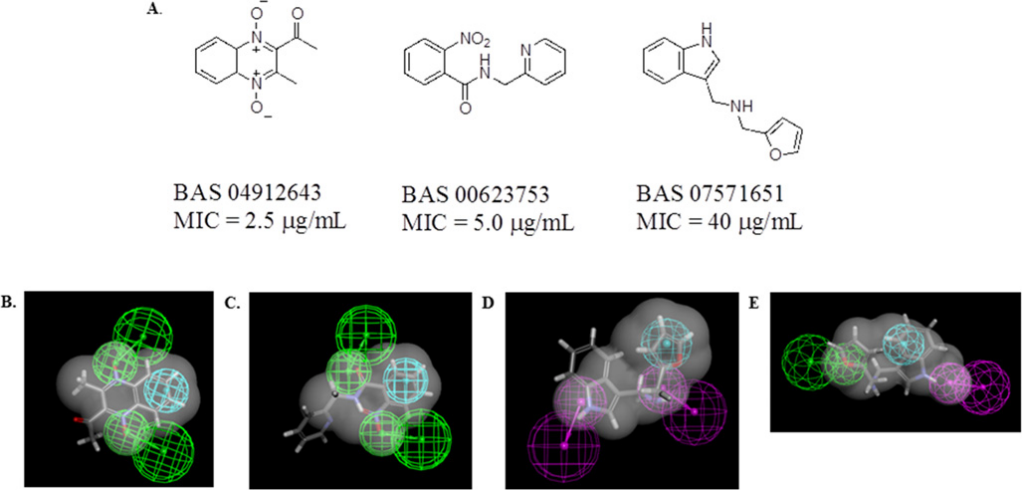
Initial pharmacophore/Bayesian model-derived hits: A) chemical structures, *in vitro* antitubercular activity, and B) best fit to menadione pharmacophore of BAS04912643, C) best fit to menadione pharmacophore of B. BAS00623753 (grey). D. best fit to indole-3-acetamide pharmacophore of BAS7571651, E best fit to lipoamide shape of BAS7571651. The pharmacophores consist of hydrogen bond acceptors (green) hydrogen bond donors (purple) and hydrophobic features (blue). The van der Waals surface was used to limit the number of compounds retrieved when screening the vendor library.

From the set of 110 compounds tested initially, three compounds (1-(3-methyl-1,4-dioxy-quinoxalin-2-yl)-ethanone [BAS 04912643], 2-nitro-N-pyridin-2-ylmethyl-benzamide [BAS 00623753] and furan-2-ylmethyl-(1H-indol-3-ylmethyl)-amine [BAS 7571651]) showed minimal inhibitory concentration (MIC) values against the *in vitro* cultured *Mtb* H37Rv strain of 2.5, 5.0 and 40 μg/mL, respectively (Fig 1A). The remaining compounds had MIC values > 40 μg/mL (data not shown). BAS04912643 and BAS00623753 mapped to the menadione pharmacophore (Fig 1B and 1C) while BAS7571651 mapped to both the lipoamide shape and indole-3-acetamide pharmacophores (Fig 1D and 1E).

### Hit exploration

**Fig 2:**
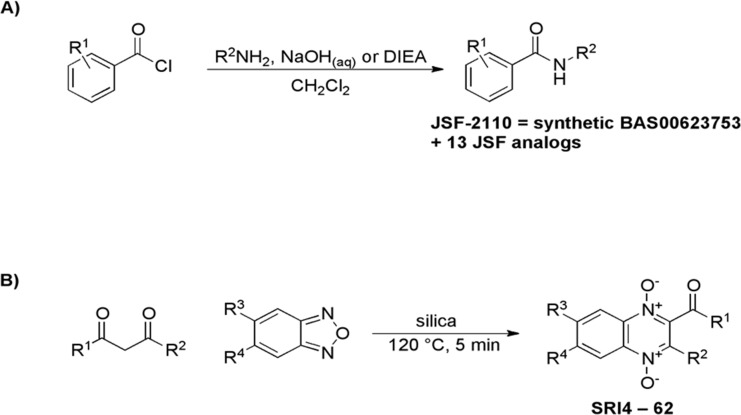
Synthetic routes to the A) arylamide and B) quinoxaline di-*N*-oxide families.

We have further explored the structure-activity relationships (SAR) for the two most potent hits. Initial efforts with BAS 00623753 consisted of the synthesis of the initial hit along with 13 analogs (Table 1, details as to the synthesis and characterization of all compounds may be found in the S1 Data). The alterations included: removal of the nitro group from the aroyl ring or its replacement with an electron-donating group (CH_3_) or other electron-withdrawing groups (F, CF_3_); α,α-dimethylation of the one-carbon tether between the amide nitrogen and the heteroaryl group or its homologation; and replacement of the 2-pyridyl moiety with differentially substituted pyridines or other heterocycles of the pyrazine and pyrimidine families. Their syntheses (Fig 2) were realized through the facile coupling of the aroyl chloride and amine partners in moderate to good yields. The small molecules were then assayed for their growth inhibition of *Mtb*. Disappointingly, the synthesized version of BAS 00623753 exhibited an MIC ≥ 50 μg/mL as did the other analogs. The original commercial sample that demonstrated promising whole-cell efficacy was no longer available and thus an analytical comparison of the two materials was not feasible.

**Table 1:** *Mtb* growth inhibitory activities of BAS 00623753 and a small set of analogs. Molecule structures are in S1 Data.

Compound #	Ar	R	*Mtb* MIC (H37Rv) μg/mL
JSF-2210 = synthetic BAS00623753	2-NO_2_Ph	CH_2_(2-pyridyl)	>50
JSF-2133	Ph	CH_2_(2-pyridyl)	>50
JSF-2170	2-NO_2_Ph	(CH_2_)_2_(2-pyridyl)	>50
JSF-2171	2-NO_2_Ph	CH_2_(2-pyrazinyl)	>50
JSF-2172	2-NO_2_Ph	CH_2_(2-(3 Mepyridyl))	>50
JSF-2173	2-NO_2_Ph	CH_2_(2-(4-Mepyridyl))	>50
JSF-2174	2-NO_2_Ph	CH_2_(2-(5-Clpyridyl))	>50
JSF-2175	2-NO_2_Ph	CH_2_(2-(5-Mepyridyl))	>50
JSF-2177	2-NO_2_Ph	CH_2_(4-pyrimidinyl)	>50
JSF-2178	2-NO_2_Ph	CH_2_(2-pyrimidinyl)	>50
JSF-2176	2-NO_2_Ph	CMe_2_(2-pyrimidinyl)	>50
JSF-2208	2-FPh	CH_2_(2-pyridyl)	>50
JSF-2209	2-MePh	CH_2_(2-pyridyl)	>50
JSF-2211	2-CF_3_Ph	CH_2_(2-pyridyl)	>50

BAS004912643 (**1**) was identified as a potential substrate mimetic of menadione (Fig 1) and demonstrated an MIC against *Mtb* of 2.5 μg/mL. To validate the hit, we developed an SAR for both antitubercular efficacy and the cytotoxicity to model mammalian (Vero) cells through determination of the CC_50_ (amount of compound to inhibit cell growth by 50%). Structural queries of the Available Chemicals Directory (ACD) (http://accelrys.com/products/databases/sourcing/available-chemicals-directory.html), SciFinder (http://www.cas.org/products/scifinder) and eMolecules (www.emolecules.com) were performed to identify structural analogs of **1** available for purchase. Of the commercially available analogs, only two compounds (quinoxaline di-*N*-oxides **2** and **3,** Table 2) were subjectively viewed as sufficiently similar while also being predicted to be whole-cell active through our Bayesian models. These analogs were purchased and tested for their antitubercular activity (Table 2). Their whole-cell efficacy was confirmed experimentally. To further establish an SAR for the quinoxaline di-*N-*oxides, we used a concise synthetic route that consisted of heating a benzofuroxan with a 2,4-pentanedione in the presence of silica gel (Fig 2). This one-step reaction gave acceptable yields (20–80%) of desired product, though often generated regioisomers depending on the nature of the benzoxadiazole *N*-oxide. The regioisomers were generally separable via flash chromatography and both isomers were tested for activities; in some instances chromatographic separation of the isomers was not achieved. This method was utilized to prepare 62 analogs that have been fully characterized via LC-MS and ^1^H NMR spectroscopy and tested for their MIC value against *Mtb* (Table 2).

**Table 2:** Structures and activities of the quinoxaline di-*N-*oxide family (nd = not determined). Molecule structures are in S1 Data.

Compound SRI#	R^1^	R^2^	R^3^	R^4^	*Mtb* MIC (H_37_Rv) μg/mL	Vero Cell CC_50_ μg/mL	% R^3^/R^4^ position regioisomers	% R^1^/R^2^ position regioisomers
**1**	CH_3_ (BAS004912643)	CH_3_	H	H	2.5	>40		
**2**	CH_3_	CH_3_	CH_3_	H	5.0	nd		
**3**	CH_3_	CH_3_	OCH_3_	H	10	nd		
**4**	CH_3_	CH_3_	H	Cl	5	>40	<10	
**5**	CH_3_	CH_3_	H	NO_2_	2.5	nd		
**6**	C_6_H_5_	CH_3_	H	CH_3_	>40	nd	70	
**7**	C_6_H_5_	CH_3_	H	Cl	5	nd	<5	
**8**	C_6_H_5_	CH_3_	Cl	H	10	nd		
**9**	C_6_H_5_	CH_3_	H	OCH_3_	5	nd	<5	
**10**	C_6_H_5_	CH_3_	H	NO_2_	5	nd	<10	
**11**	3-pyridyl	CH_3_	H	Cl	10	nd		
**12**	3-pyridyl	CH_3_	H	OCH_3_	0.64	1.7	25	
**13**	3-pyridyl	CH_3_	H	NO_2_	20	nd	50	
**14**	CF_3_	CH_3_	H	CH_3_	10	nd	38	
**15**	CF_3_	CH_3_	H	OCH_3_	10	nd	<5	
**16**	CH_2_CH_2_CH_2_CH_3_	CH_3_	H	Cl	5	nd		
**17**	CH_2_CH_2_CH_2_CH_3_	CH_3_	Cl	H	2.5	nd		
**18**	CH_2_CH(CH_3_)_2_	CH_3_	Cl	H	10	nd		
**19**	CH_2_CH(CH_3_)_2_	CH_3_	H	Cl	5	nd		
**20**	CH_2_CH_2_CH_2_CH_2_CH_3_	CH_3_	H	Cl	5	nd		
**21**	CH_2_CH_2_CH_2_CH_2_CH_3_	CH_3_	Cl	H	10	nd		
**22**	CH_2_CH_2_CHCH_2_	CH_3_	H	Cl	5	nd		
**23**	CH_2_CH_3_	CH_2_CH_3_	H	Cl	5	nd		
**24**	CH_2_CH_3_	CH_2_CH_3_	Cl	H	10	nd		
**25**	CHCH_2_CH_2_	CH_3_	H	Cl	5	nd		
**26**	CHCH_2_CH_2_	CH_3_	Cl	H	5	nd		
**27**	CH(CH_3_)_2_	CH_3_	H	Cl	>40	nd		
**28**	CH(CH_3_)_2_	CH_3_	Cl	H	10	nd		
**29**	CH_2_CH_2_CHC(CH_3_)_2_	CH_3_	H	Cl	5	nd		
**30**	CH_2_CH_2_CH_2_CH_3_	CH_3_	H	CH_3_	5	nd		
**31**	CH_2_CH_2_CH_2_CH_2_CH_3_	CH_3_	H	CH_3_	20	nd	36	
**32**	CH_2_CH_3_	CH_2_CH_3_	H	CH_3_	5	nd	40	
**33**	CHCH_2_CH_2_	CH_3_	H	CH_3_	5	nd	40	
**34**	CH_2_CH(CH_3_)_2_	CH_3_	H	CH_3_	20	nd	42	
**35**	CH_2_CH_2_CHCH_2_	CH_3_	H	CH_3_	4.3	nd	54	
**36**	CH_2_CH_2_CHC(CH_3_)_2_	CH_3_	H	CH_3_	10	nd	50	
**37**	CH_2_CH_3_	CH_3_	H	CH_3_	10	nd	25	
**38**	CH(CH_3_)_2_	CH_3_	H	CH_3_	10	nd	50	
**39**	CH_2_CH_2_CH_2_CH_3_	CH_3_	H	OCH_3_	10	nd		35
**40**	CH_2_CH_2_CH_2_CH_2_CH_3_	CH_3_	H	OCH_3_	>40	nd		25
**41**	CH_2_CH_3_	CH_2_CH_3_	H	OCH_3_	40	nd		
**42**	CH(CH_3_)_2_	CH_3_	H	OCH_3_	40	nd	<5	
**43**	CH_2_CH(CH_3_)_2_	CH_3_	H	OCH_3_	20	nd		15
**44**	CH_2_CH_2_CHCH_2_	CH_3_	H	OCH_3_	20	nd	<5	
**45**	CH_2_CH_2_CHC(CH_3_)_2_	CH_3_	H	OCH_3_	20	nd	<5	
**46**	CH_2_CH_3_	CH_3_	H	OCH_3_	20	nd	<5	
**47**	CH_2_CH_2_CHC(CH_3_)CH_2_CH_2_CHC(CH_3_)_2_	CH_3_	H	OCH_3_	20	nd		40
**48**	CH_2_CH_2_CHC(CH_3_)CH_2_CH_2_CHC(CH_3_)_2_	CH_3_	H	CH_3_	5	nd		30
**49**	CH_2_CH_2_CHC(CH_3_)CH_2_CH_2_CHC(CH_3_)_2_	CH_3_	H	Cl	5	nd		40
**50**	CH_2_CH_2_CH_2_CH_3_	CH_3_	H	NO_2_	0.32	3.4	25	
**51**	CH_2_CH_2_CHC(CH_3_)CH_2_CH_2_CHC(CH_3_)_2_	CH_3_	H	H	5	nd		50
**52**	OCH_2_CH_3_	CH_3_	H	H	>40	nd		
**53**	OH	CH_3_	H	H	>40	nd		
**54**	CH_2_CH_2_CH_2_CH_3_	CH_3_	Cl	Cl	2.5	15		25
**55**	3-pyridyl	CH_3_	Cl	Cl	2.5	>40		
**56**	CH_2_CH_2_CHC(CH_3_)CH_2_CH_2_CHC(CH_3_)_2_	CH_3_	Cl	Cl	2.5	4.5		
**57**	CH_2_CH_2_CHC(CH_3_)_2_	CH_3_	Cl	Cl	1.25	>40		
**58**	CHCH_2_CH_2_	CH_3_	Cl	Cl	1.25	>40		25
**59**	CH_2_CH_2_CH_2_CH_2_CH_3_	CH_3_	Cl	Cl	2.5	nd		30
**60**	CH_2_CH(CH_3_)_2_	CH_3_	Cl	Cl	2.5	nd		15
**61**	CH_2_CH_2_CHCH_2_	CH_3_	Cl	Cl	2.5	nd		40
**62**	C_6_H_5_	CH_3_	Cl	Cl	2.5	nd		15

A range of aliphatic groups appeared to be tolerated at R^1^ in deference to an ester (**52**) or acid (**53**) where the MIC was >40 μg/mL. A small set of substituents (H, Cl, CH_3_, OCH_3_, NO_2_) was examined at the 5- and 6- positions of the benzofuroxan input to afford final compounds with potencies that varied significantly depending on the other substituting groups in the quinoxaline. The two most potent antitubercular compounds were **50** (MIC = 0.32 μg/mL) and **12** (MIC = 0.64 μg/mL).

**Fig 3:**
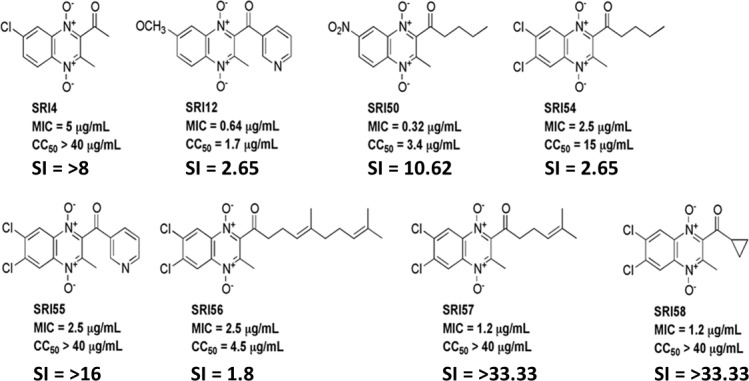
Structures of quinoxaline di-*N-*oxides with the most promising antitubercular activities and selectivities.

Eight analogs with an MIC ≤ 5.0 μg/mL, in addition to the original hit **1**, were also tested for cytotoxicity to Vero cells to assess the selectivity for antimicrobial activity relative to cytotoxicity (SI = CC_50_/MIC) (Fig 3). Three compounds exhibited an undesirable SI < 10 (**SRI12**, **SRI54**, and **SRI56**). Amongst the five satisfying this SI criterion, **SRI57** and **SRI58** both demonstrated SI > 32.

Given their promising *in vitro* activity and cytotoxicity, **SRI50** and **SRI58** were profiled for kinetic solubility in pH 7.4 PBS, mouse liver microsomal stability, and Caco-2 cell permeability (Table 3). Due to its structural similarity to these two analogs, **SRI54** was also tested in this panel. **SRI50** and **SRI58** were approximately eight-fold more soluble than **SRI54**. **SRI58** exhibited significantly greater mouse liver microsomal stability than the two other analogs. With all three quinoxalines, metabolism appeared to be NADPH-dependent. All three compounds exhibited comparatively low Caco-2 cell permeability (P_app_ < 10 x 10^−6^ cm/s) in both directions with efflux not being a significant issue (P_B-A_/P_A-B_ < 3). Poor recovery of the compounds, due to either low aqueous solubility and/or non-specific binding to the cell monolayer, may have affected the overall measurements of compound concentration on each side of the monolayer (especially those of **SRI50**, for which permeabilities could not be quantified). **SRI58** (formulation: 10% DMA/90% (20% Solutol in citrate buffer pH 3.5)) did not exhibit quantifiable blood levels in mouse pharmacokinetic studies (iv and po; data not shown).

**Table 3:** Physiochemical and ADME data. For microsomal stability, verapamil was used as a high-metabolism control (0.24% remaining with NADPH) and warfarin was a low-metabolism control (85% remaining with NADPH). The kinetic solubility limit was the highest concentration with no detectable precipitate. For Caco-2 cell permeability, compounds at a concentration of 10 μM were incubated for 2 h. P_app_ = apparent permeability coefficient. All compounds showed poor recovery due to either low solubility or non-specific binding. Ranitidine, warfarin and talindol were used as low permeability, high permeability and P-gp efflux, controls respectively.

	Mouse liver microsomal stability		Kinetic Solubility	Caco-2 Cell Permeability		
Molecule	% Compound remaining after 1h in the presence of NADPH (%)	% Compound remaining after 1h in the absence of NADPH (%)	Solubility Limit at 2 h (μM)	Mean A->B P_app_ (10^−6^ cm s^-1^)	Mean B->A Papp (10^−6^ cm s^-1^)	Efflux ratioPapp (B->A)/Papp (A->B)
**SRI50**	0.06	77.5	125	0.0	0.0	N/A
**SRI54**	0	79.1	15.6	0.66	0.10	0.15
**SRI58**	63.6	110	125	2.3	0.57	0.25

### *In vitro* activity against MDR-TB

To avoid pursuing hits modulating biological targets pertinent to known antitubercular drugs, we tested the most potent quinoxaline di-*N*-oxide **SRI50** for activity against clinical MDR-TB strains with known drug resistance profiles [29]. **SRI50** showed potent activity against clinical susceptible as well as clinical MDR-TB strains comparable to the laboratory H_37_Rv strain suggesting a novel mechanism of action for this series (Table 4).

**Table 4:** Activity of SRI50 against wild type and clinical MDR-TB strains.

Strain	Drug Resistance^a^	Strain Type	SRI50 (μg/mL)
H37Rv	None	Laboratory	0.16
210	None	Clinical	0.31
692	pan-susceptible	Clinical	0.16
91	RIF, EMB	Clinical	0.16
36	INH, RIF, EMB	Clinical	0.16
116	INH, EMB, PAS	Clinical	0.16
31	INH, RIF, EMB, KAN, SM, CAP	Clinical	0.31

^a^ RIF = rifampicin; EMB = ethambutol; INH = isoniazid; PAS = *p-*aminosalicyclic acid; KAN = kanamycin; SM = streptomycin; CAP = capreomycin

### Mechanism of action studies through transcriptional profiling

**Fig 4:**
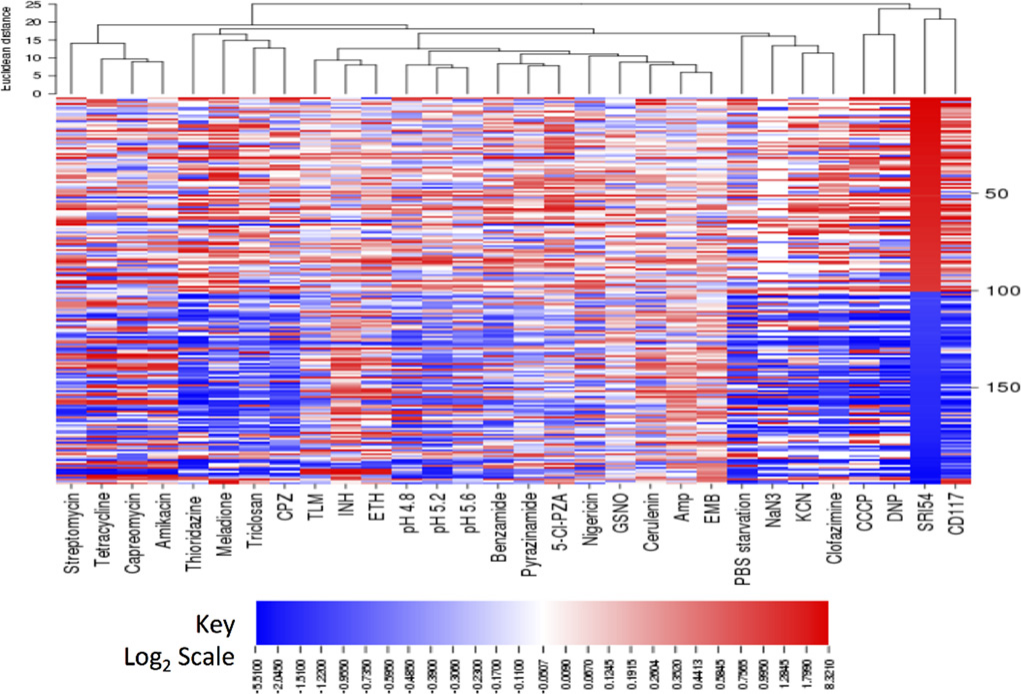
*Mtb* transcriptional response to SRI54 as compared to other small molecule antituberculars and environmental stresses. 100 SRI54 most induced and repressed genes (top-bottom) are clustered with responses to other treatments (left-right). The top dendrogram indicates relatedness of the *Mtb* perturbations based on gene clusters. Red indicates increase, blue indicates decrease and white no change in expression versus DMSO treatment. *Amp*, ampicillin; *EMB*, ethambutol; *TLM*, thiolactomycin; *INH*, isoniazid; *ETH*, ethionamide; *5-Cl-PZA*, 5-chloropyrazinamide, *CPZ*, chlorpromazine; *CCCP*, carbonyl cyanide 3-chlorophenylhydrazone; *GSNO*, S-nitrosoglutathione; *DNP*, 2,4-dinitrophenol.

To gain insight into the effect of these quinoxaline di-*N*-oxides on *Mtb*, we turned to transcriptional profiling [30, 31]. Summarily, *Mtb* grown on Middlebrook 7H9 supplemented with OADC, Tween 80, and glycerol was treated with **SRI54** at 3.2 μg/mL (1.3X MIC) for 6 h in quadruplicate and subsequently mRNA was isolated. Microarray studies (fold changes in *Mtb* genes may be found in S3 Table) allowed determination of the effects of **SRI54** on *Mtb* transcript levels as compared to a DMSO-only control. Overall, 131 genes were up-regulated at least twofold versus control and 184 genes were down-regulated at minimum twofold versus control. It is noteworthy that 3/13 genes (*prpC*, *prpD*, and *icl1*) in the methylcitrate cycle [32, 33] were induced more than fourfold versus control. The other genes (10/13) in the cycle were not significantly affected by **SRI54** as compared to control. In addition, 12/59 genes involved in DNA repair [34] were up-regulated and solely 2/59 were down-regulated at least twofold as compared to the control. Two genes in leucine biosynthesis (*leuC* and *leuD*) were down-regulated greater than equal to twofold versus control samples. These two genes encode for the two subunits of isopropylmalate dehydratase [35]. A number of genes involved in the FASII pathway [36] were similarly down-regulated, including *kasA*, *kasB*, and *InhA*. Finally, consideration of the top 100 most up-regulated and top 100 most down-regulated genes of *Mtb* when exposed to **SRI54** as compared to no-drug control through clustering with deposited *Mtb* transcriptional data (environmental stresses and small molecule antituberculars) [30] was performed via hierarchical clustering (Fig 4). It is noteworthy that **SRI54** clustered most closely to the fatty acid biosynthesis inhibitor CD117, which modulates both short-chain fatty acid and mycolic acid biosynthesis, [37, 38], and secondarily to two known protonophores, 2,4-dinitrophenol and carbonyl cyanide 3-chlorophenylhydrazone [30].

### Computational Target Prediction

It should be noted that the postulated targets for which the initial three hits were retrieved (Fig 1) are not represented in the TB Mobile Apps (version 1.0 or 2.0) used [16, 17], although they are in similar property space as the respective training sets when visualized by PCA (S2 Fig). Therefore, the predictions may suggest additional targets, which could be followed up with biochemical and/or microbiological studies. Using TB Mobile version 1.0 for BAS 04912643 the closest hit is pyrazinoic acid (S3A Fig), which is predicted to be similar to compounds that target DeaD, Mfd, RecG, DinG, and NrdR. PCA clustering also places this compound in a predominantly FabH cluster (S4 Table). For BAS00623753 the closest hit targets the UDP-galactopyranose mutase Glf (S3B Fig) while PCA clustering places it in a MurB cluster. Finally for BAS07571651 the closest hit targets Glf (S3C Fig) and PCA clustering places it in a QcrB cluster [39]. TB mobile 2.0 with its larger database, use of ECFP_6 fingerprints for similarity analysis and addition of Bayesian models for targets produced some differences in predictions (S4A Fig). For example, BAS 04912643 was predicted by the Bayesian models to target FtsZ, CysH, DprE1 and Rv1885c (S4B Fig). BAS00623753 was predicted to modulate DprE1, Rv1885, DprE2, CysH and Alr (S4C Fig). BAS07571651 was predicted to inhibit CysH and Ald (S4D Fig). When utilizing similarity criteria for each of the three hits to infer target, the following hit-target pairings were predicted: BAS04912643—CysS, BAS00623753 –Glf, and BAS07571651—InhA.

## Discussion

Combining bioinformatics data from databases (like TBCyc, SRI’s BioCyc collection [40, 41], and Pathway Logic models [42–45]) with cheminformatics databases (like CDD) as well as computational modeling approaches is rarely attempted. When it is, synergies arise which can accelerate the process of drug discovery. For example, our essential metabolite approach using 3D pharmacophore model scoring of commercial chemical space, alone [6] or in combination with our Bayesian models for antitubercular whole-cell efficacy [7], may be viewed as intermediate between high-throughput screening and rational structure-based drug design. Our previous experiments with a multi-tiered, integrative informatics workflow (using pharmacophores, Bayesian model for whole cell activity and other filters for molecular properties) identified two acylthioureas suggested as mimics of D-fructose 1,6-bisphosphate which modestly inhibited the growth of *Mtb*, and have served as a starting point for further optimization [7].

Our approach in this study differed in that we used 3D pharmacophore models and consensus amongst three dual event Bayesian models [4, 26, 27] to select compounds for testing. It produced three hits with MIC less than or equal to 40 μg/mL. If we tighten the threshold of a hit and lower it to anything less than 10 μg/mL, the two most active retrieved out of 110 represents a hit rate much lower than those we have previously described using Bayesian methods alone [46, 47]. This may be due to the far more stringent approach we have taken using multiple Bayesian models and pharmacophore models as well as other calculated properties.

Additional recent computational approaches have been proposed to select antitubercular compounds such as the druggome approach, which uses structural information on *Mtb* targets [48], although others have suggested this still requires some refinement [49]. Pharmacophores for specific *Mtb* targets have been used recently for virtual screening for acetohydroxyacid synthase inhibitors as a prefilter to docking [50]. A second study developed a pharmacophore from crystal structures for InhA and used this alongside docking to identify inhibitors [51]. Each of these cases represents the standard approach of focusing on a single target and a single pharmacophore, while in the current study we have used 66 pharmacophores representing many targets in *Mtb* to potentially identify compounds of interest from a vendor library.

With our hybrid pharmacophore-Bayesian approach, the two most active hits were retrieved by the pharmacophore based on menadione. The pharmacophore consists of two hydrogen bond acceptors and a hydrophobic feature. The enzyme NuoD (Rv3148) NADH dehydrogenase I chain D, is a subunit of NADH dehydrogenase I. The reaction catalyzed by this enzyme uses the substrate menadione and involves a complex of 13 other subunits. After testing BAS04912643 we identified that this compound–a quinoxaline 1,4-di-N-oxide–had been previously identified with an MIC of 3.13 μg/mL, possessed similar activity against resistant strains of *Mtb*, and had no appreciable mammalian cell cytotoxicity [52]. This earlier study had also shown that an analog of BAS04912643 was active *in vivo*. Further work by others has shown that quinoxaline-2-carboxylate 1,4-di-*N-*oxides have *in vitro* antitubercular activity and at least one compound was found to be active *in vivo* [53]. Similarly the closely related benzotriazine di-*N*-oxides have also been shown to have activity *in vitro* [54].

These compounds are likely bioreductively activated and we were keen to further explore the quinoxaline 1,4-di-*N*-oxide core (Table 1). **SRI50** was most potent (MIC = 0.32 μg/mL) and had a greater than 10 fold higher cytotoxicity versus Vero cells. This and other analogs (**SRI54** and **SRI58**) featuring promising *in vitro* activity and relative lack of Vero cell cytotoxicity or interesting substitutions, were then profiled for *in vitro* ADME properties. Attempts to move **SRI58** into *in vivo* infection studies in mice were halted with the failure to observe quantifiable levels of the compound in mice upon dosing iv or po. The iv result, in particular, suggests that rapid metabolism of **SRI58** may be occurring that was not observed in the *in vitro* mouse liver microsomal stability studies.

Given the lack of mechanistic information as to the *Mtb* target/s of the quinoxaline di-*N*-oxides other than our demonstration of a lack of cross-resistance of **SRI50** with front-line (isoniazid, rifampicin, ethambutol) and second-line (*p-*aminosalicyclic acid, capreomycin, streptomycin, kanamycin) drugs, we have begun to probe their mechanism of action via transcriptional profiling. Interestingly, *Mtb* treated with an early compound of interest, **SRI54** afforded a transcriptional response distinct from menadione, the *Mtb* metabolite our calculations suggested it may mimic in terms of 3D pharmacophore. The transcriptional data do, however, suggest a stress response of *Mtb* to **SRI54** exposure. SRI54 treatment resulted in up-regulation of genes (*prpC*, *prpD*, and *icl1*) within the methylcitrate cycle, reminiscent of the response of *Mtb* to isoniazid, rifampicin, and streptomycin exposure reported by the Rhee laboratory [55]. Also, potentially indicative of the *Mtb* response to **SRI54** is the up-regulation of genes involved in DNA repair. While quinaxoline di-*N*-oxides have been reported to cleave DNA through their enzymatic reduction [56], it remains to be demonstrated whether this is the result of specific damage to *Mtb* DNA by **SRI54** or a downstream consequence of the engagement of other target/s. Finally, hierarchical clustering of the transcriptional responses of *Mtb* to known antitubercular agents demonstrated a similarity of response to **SRI54**, CD117, 2,4-dinitrophenol, and carbonyl cyanide 3-chlorophenylhydrazone. It remains to be seen whether **SRI54** and other quinoxaline di-*N*-oxides inhibit *Mtb* fatty acid biosynthesis as does CD117 [37] or disrupt the proton gradient of the transmembrane electrochemical potential like 2,4-dinitrophenol and carbonyl cyanide 3-chlorophenylhydrazone [30].

We have also applied our computational approach of using Bayesian models for targets to predict the possible targets for the hits retrieved using TB Mobile [11, 16, 17]. Since the TB Mobile database does not currently include NuoD, it may be unable to predict the assumed targets of the two most active hits correctly. It does, however, suggest additional potential targets. BAS 04912643 was predicted by the Bayesian models to inhibit FtsZ, CysH, DprE1 and Rv1885c. BAS00623753 was predicted to modulate DprE1, Rv1885, DprE2, CysH and Alr. When looking at each hit and its target according to the app, the suggested target for BAS04912643 was CysS, while that from BAS00623753 was Glf. This computational approach may help prioritize targets for further testing in future. Others have recently shown how multiple computational approaches can be successfully used to predict targets that were ultimately experimentally validated [57].

In summary, we have presented the utilization of a combined bioinformatics/cheminformatics platform to arrive at candidate inhibitors of essential *Mtb* enzymes, through mimicry of the substrate/s or product/s as judged by 3D pharmacophore fit, that are predicted by a consensus amongst Bayesian models to have whole-cell efficacy. Compounds that passed the selection criteria were then tested *in vitro* versus *Mtb* and hits were validated and then optimized. We have shown clearly this strategy can lead to *in vitro* active hits that are readily synthesized (quinoxaline di-*N*-oxides), one of which had been previously shown to be an analog of a compound with both *in vitro* and *in vivo* activity [52] and potentially worthy of further evaluation because of its cost of goods. This same approach could be applied to other neglected diseases such as malaria to identify compounds with activity and the potential target/s involved. In the process of this work we have developed a computational workflow that was initially dependent on manual operation. Using the API for CDD Vault we can now also enable the automation of the computational process such that the user can go from computational target selection for pharmacophore generation to identification of molecules from vendor libraries with pharmacophores.

### Ethics Statement

Rutgers animal care and use committee approved this work, IACUC #12106A9.

## Materials and Methods

### Reagents and molecules

All experimental compounds for initial screening were purchased from Asinex (Winston-Salem, NC, USA) or synthesized in house. Purities were required to be greater than 90% with a majority of commercial compounds having a purity of greater than 95%. Compounds were all dissolved in dimethyl sulfoxide (Sigma Aldrich) at a stock concentration of 8.0 mg/mL immediately and then diluted for biological testing.

All reagents for chemical synthesis were purchased from commercial suppliers and used without further purification unless noted otherwise. All chemical reactions occurring solely in an organic solvent were carried out under an inert atmosphere of argon or nitrogen. Analytical TLC was performed with Merck silica gel 60 F_254_ plates. Silica gel column chromatography was conducted with Teledyne Isco CombiFlash Companion or Rf+ systems. ^1^H NMR spectra were acquired on Varian Inova 400, 500 and 600 MHz instruments and are listed in parts per million downfield from TMS. LC-MS was performed on an Agilent 1260 HPLC coupled to an Agilent 6120 MS. All synthesized compounds were at least 95% pure as judged by their HPLC trace at 250 nm and were characterized by the expected parent ion(s) in the MS trace. The Supplementary Materials include synthetic details pertinent to the arylamide and quinoxaline di-*N*-oxide series.

### Identification, annotation and publication of new potential *Mtb* enzyme targets

We previously described [7] in detail 1) the identification of essential *in vivo* enzymes of *Mtb*, 2) the collection of metabolic pathway and reaction information for the essential enzymes, 3) the comparison of non-human-homologous enzymes with *Mtb in vivo* essential gene set, and 4) the selection of *Mtb* targets that are essential *in vivo* but not homologous to human proteins and not known as TB drug targets.

25 *in vivo* essential enzymes (step 1) were noted in recent reports from the literature [58–61] and these include FbpC, DacB1, Cyp125, BioA, ArgJ, Nrp, SseA, End, BioF1, CobL, GcvT, AceE, HemN, AccD1, SerB2, AmiD, HsaF, Tal, FabG, NuoD, ProA, MalQ and ArcA. Among them, 2 enzymes have no human homologs (FbpC and DacB1). From a recent publication [62], we noted 32 *Mtb* enzymes with no human homologs and these are different from 66 non-human homologs found previously [7]. Except FbpC, 31 of these enzymes are not *in vivo* essential. Among these 31 non-homologous proteins, 17 are metabolic choke points [63] and 18 have the highest number of interactions with pathogenesis causing proteins. These in total give us 46 candidate essential enzyme targets among which 16 have PDB structures with a ligand bound. We have listed all these targets and annotated them (S1 Table) with respect to gene details, pathway information, structural evidence, predicted essentiality, orthologs and inhibitors information. These are supported with links to relevant databases and PubMed references. This list is published within the CDD public database (https://app.collaborativedrug.com/register) to be explored by the scientific community.

### *In silico* approaches for selecting molecules

For each molecule a 3D pharmacophore was developed using Discovery Studio 3.5 (Biovia, San Diego, CA) from 3D conformations of the substrate or metabolite. This identified key features, onto which was mapped a van der Waals surface for the molecule [3, 6, 64]. The pharmacophore plus shape was then used to search the Asinex Gold compound database (N = 205,997, for which up to 100 molecule conformations with the FAST conformer generation method with the maximum energy threshold of 20 kcal/mol, were created). The *in silico* hits were collated and uploaded in CDD, and three previously described and validated dual event Bayesian models (MLSMR, CB2 and Kinase)[8, 10, 22–25] for *Mtb* whole-cell activity were used to score the compounds and the data re-imported in CDD. All of the molecules used to build the Bayesian models are available as freely accessible datasets at www.collaborativedrug.com and Figshare [9, 23, 24]. Finally the compounds were filtered based on pharmacophore fit values > 2.5 and Bayesian scores that predicted whole-cell activity as “true”. Therefore, a compound has to comply with these criteria to be selected. Through this process 141 molecules were retrieved which was further narrowed to 110 molecules based on the opinion of an experienced medicinal chemist, removing compounds with reactive/unstable chemical functionality [28]. These compounds were then purchased for testing.

### CDD TB DB

The development of the CDD database has been described previously with applications for collaborative malaria [65] and TB research [3, 4]. The literature data on *Mtb* drug discovery has been curated and over ~20 *Mtb* specific datasets are hosted, representing well over 300,000 compounds derived from patents, literature and high throughput screening (HTS) data, and we have termed this the CDD TB DB. Some of these datasets were used to develop the Bayesian models used in this study [8]. The data generated in this study was saved in a secure CDD Vault for collaborators to share.

### CDD application programming interface development

We have described a complex workflow between different computational databases such as TBCyc and CDD Vault, as well as computational model development with Discovery Studio. To facilitate connectivity between these software packages, an application programming interface (API) was developed which allows this connectivity between software tools. The goal of this was to provide a user interface for curating TB drug targets and molecules to fully exploit published literature and data created in this project. This also integrates database searching with computational modeling tools by defining data exchange formats that enable both interactive and fully automated modeling, database searching, hit scoring and compound selection for purchasing. Ultimately this extends data types and computational modeling software capabilities upstream to target identification and validation capabilities.

The current version of TBCyc (MTH37RVV) was extended by adding drug candidates (in mol2 format) to the database via a small script of custom LISP using the Pathway Tools’ API. Associated gene/proteins were added as regulated entities of the added compounds and linked externally to a CDD Vault via the ‘**dbdef**’ and ‘**linkdef**’ command line options for linking PGDB entries with external URLs.

### Measurement of Antibacterial Activity Against *Mtb*

We used the resazurin (Alamar Blue) assay as the primary screen for activity against replicating *Mtb* [66]. Each compound was tested over a range of concentrations to determine the MIC. The antimicrobial susceptibility test was performed in a clear-bottomed, round well, 96-well microplate. Initial compounds were tested at 8 concentrations ranging between 40 and 0.31 μg/mL with a final DMSO concentration of 1.25% in each well. After a growth medium containing ~10^4^ bacteria was added to each well, the different dilutions of compounds were added. Controls included wells containing (1) concentration of rifampin and isoniazid ranging from 0.00039 to 8.0 μg/mL to control for assay performance, (2) wells with bacteria, growth medium, and vehicle (1.25% DMSO), and (3) sterility control wells with medium. Plates were incubated at 37°C for 6 d in an ambient incubator at which time 5 μL of 1% resazurin dye was added to each well. After 2 d of incubation, visual inspection of color (pink, periwinkle or blue) was recorded for each well along with measurements of fluorescence in a microplate fluorimeter with excitation at 530 nm and emission at 590 nm. The lowest drug concentration that inhibited growth of ≥90% of *Mtb* bacilli in the broth was considered the MIC value [67]. Rifampicin (MIC range 0.0031–0.012 μg/mL) and isoniazid (MIC range 0.0031–0.012 μg/mL) were used as positive controls and were consistently in the acceptable range. The MIC against MDR strains was also tested using the AlamarBlue® Cell Viability Reagent (DAL1100, ThermoFisher Scientific) as described above, except the MICs were read using absorbance as per manufacturer’s recommendation.

### Cytotoxicity determination

Vero cells (CCL-81, ATCC) were plated in 96-well plates (~5x10^4^ cells/well) and incubated overnight in cell culture media (MEM + 5% FBS + 1% Pen/strep + 1% L-Glutamine). Stock solutions of test compounds were added to cells at concentrations from 0.5–50 μM concentrations with a final DMSO concentration of 0.645% for 72 h at 37°C with 5% CO_2_. At the end of this incubation period, cell viability was measured using a Cell Titer-Glo Luminescent cell viability assay (Promega) according to manufacturer instructions. Treatment with 5% DMSO was used as a control for maximal cytotoxicity and 0.645% DMSO as a negative control. CC_50_ values were derived from plotting the calculated percent viability as a function of compound concentration and fitting the results to a four-parameter logistical function in GraphPad Prism.

### ADME/Tox Screening

With a considerable percentage of drug failures attributed to ADME/Tox (Absorption, Distribution, Metabolism, Excretion and Toxicity) issues [68, 69], it is important to assess these qualities early in the drug development process. Kinetic solubility, metabolic stability, and Caco-2 permeability were evaluated by Cyprotex (Watertown, MA).

#### Kinetic solubility

Serial dilutions of the test agent were prepared in DMSO at 100x the final concentration. Test article solutions were diluted 100-fold into pH 7.4 phosphate-buffer saline (PBS) in a 96-well plate and mixed. After 2 h at 37°C, the presence of precipitate was detected by turbidity (absorbance at 540 nm). An absorbance value of greater than ‘mean + 3x standard deviation of the blank’ (after subtracting the background) was indicative of turbidity. For brightly colored compounds, a visual inspection of the plate was performed to verify the solubility limit determined by UV absorbance. The solubility limit was reported as the highest experimental concentration with no evidence of turbidity.

#### Metabolic stability assays

The test agent was incubated in duplicate with mouse liver microsomes at 37°C. The reaction contained microsomal protein in 100 mM K_3_PO_4_, 2 mM NADPH, and 3 mM MgCl_2_ at pH 7.4. A control was run for each test agent omitting NADPH to detect NADPH-free degradation. At t = 0 and 60 min, an aliquot was removed from each experimental and control reaction and mixed with an equal volume of ice-cold Stop Solution (methanol containing propranolol as an internal standard). Stopped reactions were incubated at least ten min at -20°C, and an additional volume of water was added. The samples were centrifuged to remove precipitated protein, and the supernatants were analyzed by LC/MS/MS to quantitate the remaining parent. Data were reported as % remaining by dividing by the time zero concentration value.

#### Intestinal permeability assays

Caco-2 cells grown in tissue culture flasks were trypsinized, suspended in medium, and the suspensions were applied to wells of a Millipore 96 well Caco-2 plate. The cells were allowed to grow and differentiate for three weeks, feeding at 2 d intervals. For Apical to Basolateral (A->B) permeability, the test agent was added to the apical (A) side and amount of permeation was determined on the basolateral (B) side; for Basolateral to Apical (B->A) permeability, the test agent was added to the B side and the amount of permeation was quantified on the A side. The A-side buffer contained 100 μM Lucifer yellow dye in Transport Buffer (1.98 g/L glucose in 10 mM HEPES, 1x Hank’s Balanced Salt Solution) pH 6.5, and the B-side buffer was Transport Buffer, pH 7.4. Caco-2 cells were incubated with these buffers for 2 h, and the receiver side buffer was removed for analysis by LC/MS/MS (with propranolol used as an internal standard). To verify the Caco-2 cell monolayers were properly formed, aliquots of the cell buffers were analyzed by fluorescence to determine the transport of the impermeable dye Lucifer Yellow. Any deviations from control values were reported.

Data were expressed as permeability: (*P*_*app*_)

$$P_{app}=\frac{\text{dQ}/\text{dt}}{C_{0}A}$$

dQ/dt was the rate of permeation, C0 was initial concentration of test agent, and A was the area of monolayer.

In bidirectional permeability studies, the Efflux Ratio (R_e_) is also calculated:

$${\text{R}}_{e}=\frac{P_{app}(B\to A)}{P_{app}(A\to B)}$$

An R_e_ > 3 indicates a potential substrate for P-glycoprotein or other active transporters.

### Transcriptional Profiling

*Mtb* (H37Rv) was grown in Middlebrook 7H9 supplemented with oleic acid, albumin, dextrose, catalase, glycerol and Tween 80. At A_595_ of 0.6, cultures were treated with **SRI54** at 3.2 μg/mL (1.3X MIC) and CD117 [37] at 2 μg/mL (5X MIC) for 6 h in quadruplicates. A parallel control culture was treated with an equivalent amount of DMSO for the same amount of time. For each replicate, a total of 40 mL of *Mtb* cells (2 x 10^7^ CFU/mL) were harvested by centrifugation, homogenized with 1 mL Trizol, and transferred into a screw-cap microcentrifuge tube containing zirconia beads (0.1 mm diameter, BioSpec Products, Inc., OH). We disrupted samples by five 1 min pulses in a bead beater, keeping samples on ice for 2 min between pulses. After centrifugation, the supernatant was transferred into a clean tube and total RNA was isolated following Qiagen RNeasy mini kit. Purified RNA was kept at -80°C for further use. *Mtb* DNA microarrays were printed at the Center for Applied Genomics (CAG; http://www.cag.icph.org/) at Rutgers University [70]. The detailed labeling and hybridization protocol can be obtained at http://www.cag.icph.org/downloads_page.htm.

The 100 most induced and most repressed genes of *Mtb* in response to **SRI54** treatment were determined by mRNA expression ratio of drug-treated samples versus DMSO control samples. *Mtb* gene expression patterns under other treatments were obtained from Gene Expression Omnibus at NCBI (GEO; available on www.ncbi.nlm.nih.gov/geo) with GEO accession number GSE1642 (PMID: 15247240). These treatments included streptomycin (GSM28060), tetracycline (GSM28085), capreomycin (GSM28096), amikacin (GSM28073), PBS starvation (GSM28325), ampicillin (GSM28063), ethambutol (GSM28033), thiolactomycin (GSM28067), isoniazid (GSM28077), ethionamide (GSM28065), pH 4.8 (GSM28000), pH 5.2 (GSM28005), pH 5.6 (GSM28016), benzamide (GSM27983), pyrazinamide (GSM27898), 5-Cl-pyrazinamide (GSM27989), KCN (GSM28260), chlorpromazine (GSM28305), carbonyl cyanide 3-chlorophenylhydrazone (GSM28263), S-nitrosoglutathione (GSM28286), thioridazine (GSM28224), NaN_3_ (GSM28338), nigericin (GSM28301), clofazimine (GSM28220), 2,4-dinitrophenol (GSM28255) and menadione (GSM28307). The gene hierarchical clustering algorithm was based on the average linkage method with Euclidean distance calculation via CIMminer (discover.nci.nih.gov/cimminer) [71].

### Target predictions

Over 700 compounds with known *Mtb* targets were initially collated from the literature [7] and made available in the mobile application TB Mobile (Collaborative Drug Discovery Inc. Burlingame, CA) which is freely available for iOS and Android platforms [17, 72]. This dataset was recently updated in TB Mobile 2.0 to 805 compounds and covers 96 targets [16]. Molecules representing hits from this study were input as queries in TB Mobile versions 1 and 2.0 and the similarity of all molecules calculated in the application. A Principal Component Analysis (Discovery Studio) was also performed with all the molecules in version 1. In both versions 1 and 2 of the app, the top most structurally similar compounds (Compounds are ranked by most similar first as Tanimoto similarity is not specified in the app) were used to infer *Mtb* targets. Bayesian models integrated in the version 2.0 app were also used to predict targets. Clustering molecules with TB Mobile compounds was also undertaken in Discovery Studio (S4 Table).

## Supporting Information

S1 Data Compounds synthesized and described in Tables 1 and 2. (DOCX)

S1 Fig Pharmacophores used for database searches. (PDF)

S2 Fig PCA with the TB mobile dataset showing the 3 hits in yellow– 88.8% of variance explained in 3 PCs. (PDF)

S3 Fig TB Mobile version 1 predictions. (PDF)

S4 Fig TB Mobile 2.0 predictions. (PDF)

S1 Model Files Discovery Studio pharmacophores. (ZIP)

S1 Table Targets annotated. (XLSX)

S2 Table Targets-Reactions. (XLSX)

S3 Table S154 transcriptional profiling data. (XLSX)

S4 Table Clustering hits with TB mobile compounds (sdf file). (SDF)
