# Supplementary material for: Combining Metabolite-Based Pharmacophores with Bayesian Machine Learning Models for Mycobacterium tuberculosis Drug Discovery
Source: PLoS One. 2015 Oct 30;10(10):e0141076. doi: 10.1371/journal.pone.0141076 (PMC4627656; doi:10.1371/journal.pone.0141076)
Supplement: S1 Data — (DOCX) [file pone.0141076.s001.docx]

**PLOS ONE**

**Supplemental data**

**Combining Metabolite-Based Pharmacophores with Bayesian Machine Learning Models for *Mycobacterium tuberculosis* Drug Discovery**

Sean Ekins^1,2*^, Peter B. Madrid^3*^, Malabika Sarker^3^, Shao-Gang Li^4^, Nisha Mittal^4^, Xin Wang^4^, Thomas P. Stratton^4^, Matthew Zimmerman,^5^ Carolyn Talcott^3^, Pauline Bourbon^3^, Mike Travers^1^, Maneesh Yadav^3^ and Joel S. Freundlich^4*^

^1^Collaborative Drug Discovery Inc., 1633 Bayshore Highway, Suite 342, Burlingame, CA 94010, USA.

## ^2^Collaborations in Chemistry, 5616 Hilltop Needmore Road, Fuquay-Varina, NC 27526, USA.

^3^SRI International, 333 Ravenswood Avenue, Menlo Park, CA 94025, USA.

^4^Departments of Pharmacology & Physiology and Medicine, Center for Emerging and Reemerging Pathogens, Rutgers University – New Jersey Medical School, 185 South Orange Avenue, Newark, NJ 07103, USA.

^5^Public Health Research Institute, Rutgers University – New Jersey Medical School, Newark, NJ 07103, USA.

*Authors contributed equally; Addresses for correspondence: Sean Ekins, Collaborative Drug Discovery, 1633 Bayshore Highway, Suite 342, Burlingame, CA 94010, USA. E-mail:ekinssean@yahoo.com, Phone: 215-687-1320; Peter Madrid, SRI International, 333 Ravenswood Avenue, Menlo Park, CA 94025, USA. E-mail: [peter.madrid@sri.com](mailto:peter.madrid@sri.com); Joel S. Freundlich, Departments of Pharmacology & Physiology and Medicine, Center for Emerging and Reemerging Pathogens, Rutgers University – New Jersey Medical School, 185 South Orange Avenue Newark, NJ 07103, USA. E-mail: [freundjs@rutgers.edu](mailto:freundjs@rutgers.edu), Phone: 973-972-7165.

**Supplemental data – compounds synthesized**

**

**

Substituent format as described in Table 1

**2-nitro-N-(pyrazin-2-ylmethyl)benzamide (JSF-2171):** To 2-aminomethyl pyrazine (100 μL, 0.92 mmol) in dichloromethane (1.0 mL) was added 5% w/v NaOH (aq, 1.5 mL). 2-nitrobenzoyl chloride (109 μL, 0.92 mmol) was added dropwise and allowed to stir at rt for one hour, at which time LC-MS showed complete conversion to the intended product. The crude reaction mixture was diluted with EtOAc (10 mL), washed with water and brine (4 x 2 mL), dried over anhydrous magnesium sulfate, concentrated *in vacuo*, and chromatographed on silica gel with a 0–5% methanol/dichloromethane gradient. The product was isolated as a pale yellow solid (0.219 g**,** 85%): ^1^H NMR (500 MHz, CDCl_3_) δ 9.03 (s, 1), 8.78 (s, 1), 8.65 (s, 1), 8.09 (d, J = 8.7 Hz, 1), 7.72 (t, J = 7.4 Hz, 1), 7.62 (t, J = 6.5 Hz, 2), 7.50 (s, 1), 4.95 (d, J = 5.5 Hz, 2). (In addition, comparatively small peaks were noted at 7.9 (m), 5.32 (s, DCM), 3.19 (br s), 1.25 (s)). *m/z* (LC-MS) 259.2 [M + H]^+^.

**2-nitro-N-(2-(pyridin-2-yl)ethyl)benzamide (JSF-2170):** **JSF-2170** was obtained as an off-white solid in 80% (0.215 g) yield. ^1^H NMR (500 MHz, CDCl_3_) δ 8.54 (dd, J = 5.5, 0.9 Hz, 1), 8.16 (td, J = 7.8, 1.5 Hz, 1), 8.01 – 7.93 (m, 2), 7.71 (d, J = 7.9 Hz, 1), 7.65 (td, J = 7.5, 1.1 Hz, 1), 7.63 – 7.58 (m, 1), 7.58 – 7.52 (m, 2), 4.01 (q, J = 5.8 Hz, 2), 3.54 – 3.47 (m, 2). (In addition, comparatively small peaks were noted at 8.6 (br s), 8.25 (m), 5.32 (DCM), 4.4 (br s), 3.4 (br s), 1.3 (s), 0.8 (m).) *m/z* (LC-MS) 272.3 [M + H]^+^.

**N-((3-methylpyridin-2-yl)methyl)-2-nitrobenzamide (JSF-2172):** **JSF-2172** was obtained as an off-white solid in 80% (0.220 g) yield. ^1^H NMR (500 MHz, CDCl_3_) δ 8.75 (br s, 1), 8.51 (br s, 1), 8.11 (br s, 1), 8.03 (br s, 1), 7.78 – 7.50 (m, 4), 4.96 (br s, 2), 2.75 (d, J = 8.0 Hz, 3). (In addition, comparatively small peaks were noted at 2.6 (br s), 1.3 (br s).) *m/z* (LC-MS) 272.3 [M + H]^+^.

**N-((4-methylpyridin-2-yl)methyl)-2-nitrobenzamide (JSF-2173): JSF-2173** was obtained as a yellow wax with 75% (0.194 g) yield. ^1^H NMR (500 MHz, CDCl_3_) δ 8.73 – 8.66 (m, 1), 8.49 (d, J = 6.0 Hz, 1), 8.02 – 7.97 (m, 1), 7.87 (s, 1), 7.69 – 7.63 (m, 2), 7.62 – 7.59 (m, 1), 7.59 – 7.53 (m, 1), 4.97 (d, J = 6.5 Hz, 2), 2.68 (s, 3). (In addition, comparatively small peaks were noted at 1.24 (s), 2.5 (m), 0.8 (m).) *m/z* (LC-MS) 272.3 [M + H]^+^.

**N-((5-chloropyridin-2-yl)methyl)-2-nitrobenzamide (JSF-2174): JSF-2174** was obtained as a pale yellow solid in a 85% (0.236 g) yield. ^1^H NMR (500 MHz, CDCl_3_) δ 8.55 (d, J = 2.3 Hz, 1), 8.11 – 8.06 (m, 1), 7.92 (dd, J = 8.4, 2.4 Hz, 1), 7.71 (t, J = 7.5 Hz, 1), 7.64 – 7.59 (m, 3), 7.49 (s, 1), 4.86 (d, J = 5.5 Hz, 2). (In addition, comparatively small peaks were noted at 8.25 (m), 5.3 (s, DCM), 4.8 (m), 3.31 (br s), 2.2 (s), 2.1 (s), 1.3 (s).) *m/z* (LC-MS) 292.7 [M + H]^+^.

**N-((5-methylpyridin-2-yl)methyl)-2-nitrobenzamide (JSF-2175):** **JSF-2175** was obtained as a white solid with a 85% (0.208 g) yield. ^1^H NMR (500 MHz, CDCl_3_) δ 8.66 – 8.57 (m, 1), 8.49 (s, 1), 8.21 (d, J = 8.1 Hz, 1), 8.03 (d, J = 8.0 Hz, 1), 7.99 (d, J = 8.1 Hz, 1), 7.74 – 7.64 (m, 2), 7.63 – 7.55 (m, 1), 5.01 (d, J = 6.5 Hz, 2), 2.59 (s, 3). (In addition, comparatively small peaks were noted at 7.8 (m), small singlets 2.0 – 2.5, 1.3 (s), 0.9 (m), 0.8 (m).) *m/z* (LC-MS) 272.3 [M + H]^+^.

**2-nitro-N-(2-(pyrimidin-2-yl)propan-2-yl)benzamide (JSF-2176):** **JSF-2176** was obtained as a colorless oil in 80% (0.204 g) yield. ^1^H NMR (500 MHz, CDCl_3_) δ 8.79 (d, J = 4.9 Hz, 2), 8.08 (d, J = 8.0 Hz, 1), 7.97 (br s, 1), 7.76 – 7.68 (m, 2), 7.62 – 7.55 (m, 1), 7.30 (t, J = 4.9 Hz, 1), 1.99 (s, 6). (In addition, comparatively small peaks were noted at 5.3 (DCM), 2.8 (s), 2.2 (s), 2.1 (s), 1.8 (s), 1.6 (s), 1.3 (s), 1.2 (s), 1.1 (s).) *m/z* (LC-MS) 287.3 [M + H]^+^.

**2-nitro-N-(pyrimidin-4-ylmethyl)benzamide (JSF-2177):** **JSF-2177** was obtained as a yellow solid in 85% (0.188 g) yield. ^1^H NMR (600 MHz, d_6_-DMSO) δ 9.43 – 9.36 (m, 1), 9.14 (d, J = 1.3 Hz, 1), 8.81 (d, J = 5.2 Hz, 1), 8.07 (d, J = 8.1 Hz, 1), 7.86 – 7.81 (m, 1), 7.78 – 7.70 (m, 2), 7.55 (d, J = 5.2 Hz, 1), 4.54 (d, J = 5.9 Hz, 2). (In addition, comparatively small peaks were noted at 5.7 (s, DCM), 3.3 (s, H_2_O).) *m/z* (LC-MS) 259.2 [M + H]^+^.

**2-nitro-N-(pyrimidin-2-ylmethyl)benzamide (JSF-2178):** **JSF-2178** was obtained as a white solid in 85% (0.193 g) yield. ^1^H NMR (600 MHz, d6-acetone) δ 8.78 (d, J = 4.9 Hz, 2), 8.03 (d, J = 8.0 Hz, 1), 7.86 – 7.79 (m, 3), 7.76 – 7.70 (m, 1), 7.38 (t, J = 4.9 Hz, 1), 4.78 (s, 2). (In addition, comparatively small peaks were noted at 8.2 (br s), 5.3 (s, DCM), 2.8 (d, H_2_O), 1.3 (m), 0.8 (m), 0.1 (s).) *m/z* (LC-MS) 259.2 [M + H]^+^.

**2-fluoro-N-(pyridin-2-ylmethyl)benzamide (JSF-2208**): **JSF-2208** was obtained as a white solid in 95% (0.218 g) yield. ^1^H NMR (600 MHz, d_6_-DMSO) δ 8.91 (br s, 1), 8.52 (d, *J* = 4.4 Hz, 1), 7.83 – 7.75 (m, 1), 7.74 – 7.67 (m, 1), 7.61 – 7.51 (m, 1), 7.37 (d, *J* = 8.0 Hz, 1), 7.35 – 7.25 (m, 3), 4.57 (d, *J* = 5.9 Hz, 2). (In addition, a comparatively small peak was noted at 3.3 (s, H_2_O).) *m/z* (LC-MS) 231.2 [M + H]^+^.

**2-methyl-N-(pyridin-2-ylmethyl)benzamide (JSF-2209**): **JSF-2209** was obtained as an off-white solid in 80% (0.181 g) yield. ^1^H NMR (600 MHz, d_6_-DMSO) δ 8.87 – 8.78 (m, 1), 8.51 (d, *J* = 4.5 Hz, 1), 7.83 – 7.75 (m, 1), 7.50 (d, 1), 7.39 – 7.32 (m, 2), 7.29 – 7.22 (m, 3), 4.53 (d, *J* = 6.0 Hz, 2), 2.36 (s, 3). (In addition, a comparatively small peak was noted at 3.3 (s, H_2_O).) *m/z* (LC-MS) 227.2 [M + H]^+^.

**2-nitro-N-(pyridin-2-ylmethyl)benzamide (JSF-2210**): **JSF-2210** was obtained as an off-white solid in 80% (0.181 g) yield. ^1^H NMR (600 MHz, d_6_-DMSO) δ 9.35 – 9.26 (m, 1), 8.52 (d, *J* = 4.4 Hz, 1), 8.05 (d, *J* = 8.2 Hz, 1), 7.88 – 7.77 (m, 2), 7.76 – 7.67 (m, 2), 7.44 (d, *J* = 7.9 Hz, 1), 7.34 – 7.25 (m, 1), 4.54 (d, *J* = 6.0 Hz, 2). (In addition, a comparatively small peak was noted at 3.3 (s, H_2_O).) *m/z* (LC-MS) 258.2 [M + H]^+^.

**N-(pyridin-2-ylmethyl)-2-(trifluoromethyl)benzamide (JSF-2211**): **JSF-2211** was obtained as an off-white solid in 75% (0.210 g) yield. ^1^H NMR (600 MHz, d_6_-DMSO) δ 9.17 – 9.04 (m, 1), 8.52 (d, *J* = 4.4 Hz, 1), 7.84 – 7.78 (m, 2), 7.77 – 7.72 (m, 1), 7.69 – 7.65 (m, 1), 7.63 (d, *J* = 7.6 Hz, 1), 7.38 (d, *J* = 7.8 Hz, 1), 7.32 – 7.26 (m, 1), 4.53 (d, *J* = 6.0 Hz, 2). (In addition, a comparatively small peak was noted at 3.3 (s, H_2_O).) *m/z* (LC-MS) 281.1 [M + H]^+^.

**General Procedure for quinoxaline di*-N*-oxides syntheses**.

A benzofuroxane (0.3 mmol) and 3 g of silica gel was added to a solution of 1,3-diketone (0.3 mmol) in 10 mL of DCM. After evaporation to dryness, the mixture was transferred into a sealed microwave tube and heated for 120 s at 130 °C. The product was purified by flash chromatography on a Biotage Isolera system using a gradient of 10-60% EtOAc in hexanes, then triturated with diethyl ether and filtered to obtain the desire product. Note: yields given are for final isolated materials and poor yields were generally due to challenging chromatographic separations of regioisomers and collection of only the most pure fractions for biological testing.

**

**

Substituent format as described in Table 2

**2-acetyl-3-methylquinoxaline 1,4-dioxide (SRI1):** was obtained as an orange solid in 28% yield. ^1^H NMR (400 MHz, CDCl_3_) δ 8.60 (m, 2), 7.87 (m, 2), 2.73 (s, 3), 2.53 (s, 3). *m/z* (LC-MS) 219.12 [M + H]^+^.

**3-acetyl-2,6-dimethylquinoxaline 1,4-dioxide (SRI2):** was obtained as an orange solid in 32% yield. ^1^H NMR (400 MHz, CDCl_3_) δ 8.50 (d, J = 8.8 Hz, 1), 8.33 (s, 1), 7.50 (dd, J = 1.6 Hz, J = 8.8 Hz, 1), 2.72 (s, 3), 2.62 (s, 3), 2.52 (s, 3). *m/z* (LC-MS) 233.15 [M + H]^+^.

**3-acetyl-6-methoxy-2-methylquinoxaline 1,4-dioxide (SRI3):** was obtained as an orange solid in 45% yield. ^1^H NMR (400 MHz, CDCl_3_) δ 8.51 (d, J = 9.6 Hz, 1), 7.83 (d, J = 2.8 Hz, 1), 7.45 (dd, J = 2 Hz, J = 9.2 Hz, 1), 4.00 (s, 3), 2.73 (s, 3), 2.51 (s, 3). *m/z* (LC-MS) 249.14 [M + H]^+^.

**2-acetyl-6-chloro-3-methylquinoxaline 1,4-dioxide (SRI4):** was obtained as an orange solid in 30% yield. ^1^H NMR (400 MHz, CDCl_3_) δ 8.63 (m, 2), 7.81 (dd, J = 2.0 Hz, J = 9.2 Hz, 1), 2.72 (s, 3), 2.52 (s, 3). *m/z* (LC-MS) 253.12 [M + H]^+^.

**2-acetyl-3-methyl-6-nitroquinoxaline 1,4-dioxide (SRI5):** was obtained as an orange solid in 21% yield. ^1^H NMR (400 MHz, CDCl_3_) δ 9.48 (d, J = 2.0 Hz, 1), 8.75 (d, J = 9.2 Hz, 1), 7.45 (dd, J = 2.0 Hz, J = 9.2 Hz, 1), 2.75 (s, 3), 2.57 (s, 3). *m/z* (LC-MS) 264.10 [M + H]^+^.

**2-benzoyl-3,6-dimethylquinoxaline 1,4-dioxide (SRI6):** was obtained as an orange solid in 45% yield. ^1^H NMR (400 MHz, CDCl_3_) δ 8.57 (d, J = 8.8 Hz, 1), 8.39 (s, 1), 7.88 (d, J = 7.2 Hz, 2), 7.67 (m, 3), 7.58 (t, J =7.6 Hz, 1), 2.61 (s, 3), 2.49 (s, 3). *m/z* (LC-MS) 315.11 [M + H]^+^.

**2-benzoyl-6-chloro-3-methylquinoxaline 1,4-dioxide (SRI7):** was obtained as an orange solid in 12% yield. ^1^H NMR (400 MHz, CDCl_3_) δ 8.55 (d, J = 9.2 Hz, 1), 8.38 (d, J = 2.0 Hz, 1), 8.07 (m, 3), 7.78 (t, J = 7.6 Hz, 1), 7.58 (t, J =7.6 Hz, 2), 2.29 (s, 3). *m/z* (LC-MS) 315.11 [M + H]^+^.

**3-benzoyl-6-chloro-2-methylquinoxaline 1,4-dioxide (SRI8):** was obtained as an orange solid in 10% yield. ^1^H NMR (400 MHz, CDCl_3_) δ 8.53 (d, J =2.0 Hz, 1), 8.39 (d, J =9.2 Hz, 1), 8.07 (dd, J = 1.2 Hz, J = 9.2 Hz, 2), 8.01 (d, J = 2Hz, 9.2 Hz, 1), 7.78 (t, J = 7.6 Hz, 1), 7.58 (t, J = 7.6 Hz, 2), 2.30 (s, 3). *m/z* (LC-MS) 315.11 [M + H]^+^.

**2-benzoyl-6-methoxy-3-methylquinoxaline 1,4-dioxide (SRI9):** was obtained as an orange solid in 50 % yield. ^1^H NMR (400 MHz, CDCl_3_) δ 8.57 (d, J = 9.6 Hz, 1), 7.89 (d, J = 8.0 Hz, 2), 8.85 (d, J = 2.4 Hz, 1), 7.68 (t, J = 8.0 Hz, 1), 7.50 (m, 3), 3.98 (s, 3), 2.47 (s, 3). *m/z* (LC-MS) 315.15 [M + H]^+^.

**2-benzoyl-3-methyl-6-nitroquinoxaline 1,4-dioxide (SRI10):** was obtained as an orange solid in 30 % yield. ^1^H NMR (400 MHz, CDCl_3_) δ 9.53 (d, J = 2.0 Hz, 1), 8.74 (d, J = 9.2 Hz, 1), 8.60 (dd, J = 2 Hz, J = 9.6 Hz, 1), 7.90 (dd, J = 1.2, J = 8.0 Hz, 2) 7.72 (t, J = 7.6 Hz, 1), 7.55 (t, J = 7.6 Hz, 2), 2.53 (s, 3). *m/z* (LC-MS) 326.13 [M + H]^+^.

**6-chloro-3-methyl-2-nicotinoylquinoxaline 1,4-dioxide (SRI11):** was obtained as an orange solid in 33% yield. ^1^H NMR (400 MHz, CDCl_3_) δ 9.20 (d, J = 1.6 Hz, 1), 8.90 (dd, J = 2.0 Hz, J = 4.8 Hz, 1), 8.54 (d, J = 9.2 Hz, 1), 8.44 (dt, J = 1.6 Hz, J = 8.0 Hz, 1) 8.38 (d, J = 2.0 Hz, 1), 8.06 (dd, J = 2.4 Hz, J = 9.2 Hz, 1), 7.63 (m, 1), 2.34 (s, 3). *m/z* (LC-MS) 316.12 [M + H]^+^.

**6-methoxy-3-methyl-2-nicotinoylquinoxaline 1,4-dioxide (SRI12):** was obtained as an orange solid in 23% yield. ^1^H NMR (400 MHz, CDCl_3_) δ 9.02 (d, J = 0.8 Hz, 1), 8.87 (dd, J = 2.0 Hz, J = 4.8 Hz, 1), 8.58 (d, J = 9.6 Hz, 1), 8.21 (dt, J = 1.6 Hz, J = 8.0 Hz, 1) 7.81 (d, J = 2.8 Hz, 1), 7.50 (m, 3), 3.99 (s, 3), 2.50 (s, 3). *m/z* (LC-MS) 312.16 [M + H]^+^.

**3-methyl-2-nicotinoyl-6-nitroquinoxaline 1,4-dioxide (SRI13):** was obtained as an orange solid in 34% yield. ^1^H NMR (400 MHz, CDCl_3_) δ 9.39 (d, J = 2.4 Hz, 1), 9.36 (s, 1), 9.05 (s, 1), 8.88 (m, 2), 8.68 (dd, J = 2.4 Hz, J = 9.2 Hz, 1) 8.24 (t, J = 7.6 Hz, 1), 7.52 (m, 1), 2.55 (s, 3). *m/z* (LC-MS) 327.10 [M + H]^+^.

**3,6-dimethyl-2-(2,2,2-trifluoroacetyl)quinoxaline 1,4-dioxide (SRI14):** was obtained as an orange solid in 15% yield. ^1^H NMR (400 MHz, CDCl_3_) δ 8.50 (d, J = 8.8 Hz, 1), 8.36 (s, 1), 7.78 (d, J = 8.8 Hz, 1), 3.66 (s, 3). *m/z* (LC-MS) 287.10 [M + H]^+^.

**6-methoxy-3-methyl-2-(2,2,2-trifluoroacetyl)quinoxaline 1,4-dioxide (SRI15):** was obtained as an orange solid in 10% yield. ^1^H NMR (400 MHz, CDCl_3_) δ 8.50 (d, J = 9.6 Hz, 1), 7.83 (d, J = 2.8 Hz, 1), 7.50 (dd, J = 2.8 Hz, J = 9.6 Hz, 1), 4.08 (s, 3), 2.69 (s, 3). *m/z* (LC-MS) 303.7 [M + H]^+^.

**6-chloro-3-methyl-2-pentanoylquinoxaline 1,4-dioxide (SRI16):** was obtained as an orange solid in <5% yield. ^1^H NMR (400 MHz, CDCl_3_) δ 8.57 (m, 2), 7.80 (dd, J =2.8 Hz, J = 9.6 Hz, 1), 2.97 (t, J = 9.6 Hz, 2), 2.48 (s, 3), 1.76 (q, J = 10.4 Hz, 2), 1.45 (q, J = 10.0 Hz, 2), 0.95 (t, J = 10.0 Hz, 3). *m/z* (LC-MS) 295.16 [M + H]^+^.

**6-chloro-2-methyl-3-pentanoylquinoxaline 1,4-dioxide (SRI 17):** was obtained as an orange solid in 5% yield. ^1^H NMR (400 MHz, CDCl_3_) δ 8.57 (d, J = 2.8 Hz, 1), 8.49 (d, J = 12 Hz, 1), 7.78 (d, J = 2.8 Hz, J =12.0 Hz, 1), 2.99 (t, J =10.0 Hz, 2), 2.51 (s, 3), 1.77 (q, J = 10.4 Hz, 2), 1.46 (q, J = 10 Hz, 2), 0.96 (t, J = 10 Hz, 3). *m/z* (LC-MS) 295.16 [M + H]^+^.

**6-chloro-2-methyl-3-(3-methylbutanoyl)quinoxaline 1,4-dioxide (SRI 18):** was obtained as an orange solid in 11% yield. ^1^H NMR (400 MHz, CDCl_3_) δ 8.57 (m, 2), 7.80 (dd, J = 2.8 Hz, J = 9.6 Hz, 1), 2.88 (d, J = 8.8 Hz, 2), 2.35 (m, 1), 1.05 (d, J = 8.8Hz, 6). *m/z* (LC-MS) 295.16 [M + H]^+^.

**6-chloro-3-methyl-2-(3-methylbutanoyl)quinoxaline 1,4-dioxide (SRI19):** was obtained as an orange solid in 12% yield. ^1^H NMR (400 MHz, CDCl_3_) δ 8.64 (s, 1), 8.52 (d, J = 12.0 Hz, 1), 7.78 (d, J = 0.8 Hz, J = 12.0 Hz, 1), 2.88 (d, J = 8.8 Hz, 2), 2.35 (m, 1), 1.05 (d, J = 8.8Hz, 6). *m/z* (LC-MS) 295.10 [M + H]^+^.

**6-chloro-2-hexanoyl-3-methylquinoxaline 1,4-dioxide (SRI20):** was obtained as an orange solid in 5% yield. ^1^H NMR (400 MHz, CDCl_3_) δ 8.57 (m, 2), 7.80 (dd, J = 2.8 Hz, J = 9.6 Hz, 1), 2.97 (t, J = 10.4 Hz, 2), 2.48 (s, 3), 1.78 (q, J = 10.4 Hz, 2), 1.37(m, 4), 0.91 (t, J = 8.4 Hz, 3). *m/z* (LC-MS) 309.14 [M + H]^+^.

**6-chloro-3-hexanoyl-2-methylquinoxaline 1,4-dioxide (SRI21):** was obtained as an orange solid in <5% yield. ^1^H NMR (400 MHz, CDCl_3_) δ 8.57 (d, J = 2.8 Hz, 1), 8.49 (d, J = 12.0 Hz, 1), 7.78 (d, J = 2.8 Hz, J = 12.0 Hz, 1), 2.97 (t, J = 10.4 Hz, 2), 2.51 (s, 3), 1.78 (q, J = 9.6 Hz, 2), 1.38 (m, 4), 0.92 (t, J = 8.4 Hz, 3). *m/z* (LC-MS) 309.16 [M + H]^+^.

**6-chloro-3-methyl-2-(pent-4-enoyl)quinoxaline 1,4-dioxide (SRI22):** was obtained as an orange solid in 2% yield. ^1^H NMR (400 MHz, CDCl_3_) δ 8.57 (m, 2), 7.80 (dd, J =2.8 Hz, J = 9.6 Hz, 1), 5.82 (m, 1), 5.05 (m, 2), 2.98 (m, 2), 2.50 (m, H). *m/z* (LC-MS) 293.10 [M + H]^+^.

**6-chloro-3-ethyl-2-propionylquinoxaline 1,4-dioxide (SRI23):** was obtained as an orange solid in 16 % yield. ^1^H NMR (400 MHz, CDCl_3_) δ 8.57 (m, 2), 7.80 (dd, J = 2.8 Hz, J = 9.6 Hz, 1), 2.98 (m, 2), 2.85 (m, 2), 1.29 (m, 6). *m/z* (LC-MS) 281.14 [M + H]^+^.

**6-chloro-2-ethyl-3-propionylquinoxaline 1,4-dioxide (SRI24):** was obtained as an orange solid in <5% yield. ^1^H NMR (400 MHz, CDCl_3_) δ 8.63 (s, 1), 8.48 (dd, J = 3.2 Hz, J = 12.0 Hz, 1), 7.78 (dd, J = 3.2 Hz, J = 12.0 Hz, 1), 2.99 (m, 2), 2.87 (m, 2), 1.30 (m, 6). *m/z* (LC-MS) 281.14 [M + H]^+^.

**6-chloro-2-(cyclopropanecarbonyl)-3-methylquinoxaline 1,4-dioxide (SRI25):** was obtained as an orange solid in <5% yield. ^1^H NMR (400 MHz, CDCl_3_) δ 8.57 (m, 2), 7.80 (dd, J =2.8 Hz, J = 9.6 Hz, 1), 2.50 (m, 4), 1.44 (m, 2), 1.25 (m, 2). *m/z* (LC-MS) 279.15 [M + H]^+^.

**6-chloro-3-(cyclopropanecarbonyl)-2-methylquinoxaline 1,4-dioxide (SRI 26):** was obtained as an orange solid in 21% yield. ^1^H NMR (400 MHz, CDCl_3_) δ 8.64 (s, 1), 8.52 (d, J = 12 Hz, 1), 7.78 (d, J = 0.8 Hz, J = 12.0 Hz, 1), 2.53 (m, 4), 1.45 (m, 2), 1.26 (m, 2). *m/z* (LC-MS) 279.15 [M + H]^+^.

**6-chloro-2-isobutyryl-3-methylquinoxaline 1,4-dioxide (SRI27):** was obtained as an orange solid in <5% yield. ^1^H NMR (400 MHz, CDCl_3_) δ 8.57 (m, 2), 7.82 (dd, J =2.8 Hz, J = 9.6 Hz, 1), 3.48 (m, 1), 1.28 (m, 6). *m/z* (LC-MS) 281.13 [M + H]^+^.

**6-chloro-3-isobutyryl-2-methylquinoxaline 1,4-dioxide (SRI28):** was obtained as an orange solid in <5% yield. ^1^H NMR (400 MHz, CDCl_3_) δ 8.65 (s, 1), 8.50 (d, J = 12.0 Hz, 1), 7.78 (d, J = 0.8 Hz, J = 12.0 Hz, 1), 3.42 (m, 1), 1.28 (m, 6). *m/z* (LC-MS) 281.15 [M + H]^+^.

**6-chloro-3-methyl-2-(5-methylhex-4-enoyl)quinoxaline 1,4-dioxide (SRI29):** was obtained as an orange solid in <5% yield. ^1^H NMR (400 MHz, CDCl_3_) δ 8.55 (m, 2), 7.80 (dd, J = 2.8 Hz, J = 9.6 Hz, 1), 5.18 (m, 1), 2.91 (t, J = 10.4 Hz, 2), 2.47 (m, 2), 1.68 (s, 3), 1.56 (s, 3). *m/z* (LC-MS) 312.11 [M + H]^+^.

**3,6-dimethyl-2-pentanoylquinoxaline 1,4-dioxide (SRI30):** was obtained as an orange solid in <5% yield. ^1^H NMR (400 MHz, CDCl_3_) δ 8.50 (d, J = 12.0 Hz, 1), 8.33 (s, 1), 7.69 (d, J = 12.0 Hz, 1), 2.99 (t, J = 10.0 Hz, 2), 2.61 (s, 3), 2.48 (s, 3), 1.77 (q, J = 10.4 Hz, 2), 1.46 (q, J = 10.0 Hz, 2), 0.96 (t, J = 10.0 Hz, 3). *m/z* (LC-MS) 275.19 [M + H]^+^.

**2-hexanoyl-3,6-dimethylquinoxaline 1,4-dioxide (SRI31):** was obtained as an orange solid in <5% yield. ^1^H NMR (400 MHz, CDCl_3_) δ 8.45 (m, 2), 7.69 (m, 1), 2.97 (t, J = 10.4 Hz, 2), 2.60 (s, 3), 2.50 (s, 3), 1.78 (q, J = 10.4 Hz, 2), 1.37 (m, 4), 0.91 (t, J = 8.4 Hz, 3). *m/z* (LC-MS) 289.19 [M + H]^+^.

**3-ethyl-6-methyl-2-propionylquinoxaline 1,4-dioxide (SRI32):** was obtained as an orange solid in 10 % yield. ^1^H NMR (400 MHz, CDCl_3_) δ 8.57 (m, 2), 7.65 (m, 1), 2.99 (m, 2), 2.85 (m, 2), 1.29 (m, 6). *m/z* (LC-MS) 261.15 [M + H]^+^.

**2-(cyclopropanecarbonyl)-3,6-dimethylquinoxaline 1,4-dioxide (SRI33):** was obtained as an orange solid in 21% yield. ^1^H NMR (400 MHz, CDCl_3_) δ 8.51 (m, 2), 7,66 (m, 1), 2.62 (s, 3), 2.52 (m, 1), 1.42 (m, 2), 1.22 (m, ). *m/z* (LC-MS) 259.13 [M + H]^+^.

**3,6-dimethyl-2-(3-methylbutanoyl)quinoxaline 1,4-dioxide (SRI34):** was obtained as an orange solid in 17% yield. ^1^H NMR (400 MHz, CDCl_3_) δ 8.50 (s, 1), 8.38 (d, J = 12.0 Hz, 1), 7.65 (m, 1), 2.89 (d, J = 8.8 Hz, 2), 2.60 (s, 3), 2.35 (m, 1), 1.05 (d, J = 8.8 Hz, 6). *m/z* (LC-MS) 275.18 [M + H]^+^.

**3,6-dimethyl-2-(pent-4-enoyl)quinoxaline 1,4-dioxide (SRI35):** was obtained as an orange solid in <5% yield. ^1^H NMR (400 MHz, CDCl_3_) δ 8.44 (m, 2), 7.65 (d, J = 9.6 Hz, 1), 5.82 (m, 1), 5.05 (m, 2), 2.98 (m, 2), 2.72 (s, 3), 2.50 (m, 2). *m/z* (LC-MS) 273.15 [M + H]^+^.

**3,6-dimethyl-2-(5-methylhex-4-enoyl)quinoxaline 1,4-dioxide (SRI36):** was obtained as an orange solid in 10% yield. ^1^H NMR (400 MHz, CDCl_3_) δ 8.40 (m, 2), 7.65 (m, 1), 5.18 (m, 1), 3.03 (t, J = 10.4 Hz, 2), 2.68 (s, 3), 2.47 (m, 2), 1.68 (s, 3), 1.56 (s, 3). *m/z* (LC-MS) 301.14 [M + H]^+^.

**2,6-dimethyl-3-propionylquinoxaline 1,4-dioxide (SRI37):** was obtained as an orange solid in <5% yield. ^1^H NMR (400 MHz, CDCl_3_) δ 8.48 (d, J = 12.0 Hz, 1), 8.32 (s, 1), 7.65 (d, J = 12.0 Hz, 1), 2.99 (m, 2), 2.61 (s, 3), 2.51 (s, 3), 1.29 (t, J = 10.0 Hz, 3). *m/z* (LC-MS) 247.13 [M + H]^+^.

**2-isobutyryl-3,6-dimethylquinoxaline 1,4-dioxide (SRI38):** was obtained as an orange solid in 7% yield. ^1^H NMR (400 MHz, CDCl_3_) δ 8.41 (m, 2), 7.67 (m 1), 3.41 (m, 1), 2.63 (s, 3), 2.49 (s, 3), 1.28 (m, 6). *m/z* (LC-MS) 261.13 [M + H]^+^.

**6-methoxy-2-methyl-3-pentanoylquinoxaline 1,4-dioxide (SRI39):** was obtained as an orange solid in <5% yield. ^1^H NMR (400 MHz, CDCl_3_) δ 8.49 (d, J = 12.8 Hz, 1), 7.81 (s, 1), 7.45 (d, J = 12.8 Hz, 1), 2.99 (t, J = 10.0 Hz, 2), 2.47 (s, 3), 1.77 (m, 2), 1.46 (m, 2), 0.95 (t, J = 10.0 Hz, 3). *m/z* (LC-MS) 291.17 [M + H]^+^.

**3-hexanoyl-6-methoxy-2-methylquinoxaline 1,4-dioxide (SRI40):** was obtained as an orange solid in <5% yield. ^1^H NMR (400 MHz, CDCl_3_) δ 8.52 (d, J = 12.4 Hz, 1), 7.82 (s, 1), 7.45 (d, J = 12.4 Hz, 1), 4.00 (s, 3), 2.98 (t, J = 10.4 Hz, 2), 2.48 (s, 3), 1.78 (m, 2), 1.37 (m, 4), 0.91 (t, J = 8.4 Hz, 3H). *m/z* (LC-MS) 305.15 [M + H]^+^.

**2-ethyl-6-methoxy-3-propionylquinoxaline 1,4-dioxide (SRI41):** was obtained as an orange solid in <5% yield. ^1^H NMR (400 MHz, CDCl_3_) δ 8.51 (d, J = 12.4 Hz, 1), 7.80 (s, 1), 7.42 (d, J = 12.4 Hz, 1), 2.99 (q, J = 9.6 Hz, 2), 2.84 (q, J = 9.6 Hz, 2), 1.30 (t, J = 9.6 Hz, 6). *m/z* (LC-MS) 277.12 [M + H]^+^.

**3-(cyclopropanecarbonyl)-6-methoxy-2-methylquinoxaline 1,4-dioxide (SRI42):** was obtained as an orange solid in <5% yield. ^1^H NMR (400 MHz, CDCl_3_) δ 8.51 (d, J = 12.4 Hz, 1), 7.80 (s, 1), 7.42 (d, J = 12.4 Hz, 1), 3.98 (s, 3), 2.49 (m, 1), 1.43 (m, 2), 1.22 (m, ). *m/z* (LC-MS) 275.12 [M + H]^+^.

**6-methoxy-2-methyl-3-(3-methylbutanoyl)quinoxaline 1,4-dioxide (SRI43):** was obtained as an orange solid in <5% yield. ^1^H NMR (400 MHz, CDCl_3_) δ 8.50 (d, J = 12.4 Hz, 1), 7.80 (s, 1), 7.42 (d, J = 12.4 Hz, 1), 3.89 (s, 3), 2.89 (d, J = 8.8 Hz, 2), 2.47 (s, 3), 2.39 (m, 1), 1.05 (d, J = 8.8 Hz, 6). *m/z* (LC-MS) 291.16 [M + H]^+^.

**6-methoxy-2-methyl-3-(pent-4-enoyl)quinoxaline 1,4-dioxide (SRI44):** was obtained as an orange solid in <5% yield. ^1^H NMR (400 MHz, CDCl_3_) δ 8.52 (d, J = 12.4 Hz, 1), 7.83 (s, 1), 7.46 (d, J = 12.4 Hz, 1), 5.04 (m, 2), 4.00 (s, 3), 2.96 (m, 2), 2.72 (s, 3), 2.47 (m, 2). *m/z* (LC-MS) 289.14 [M + H]^+^.

**6-methoxy-2-methyl-3-(5-methylhex-4-enoyl)quinoxaline 1,4-dioxide (SRI45):** was obtained as an orange solid in <5% yield. ^1^H NMR (400 MHz, CDCl_3_) δ 8.44 (d, J = 12.4 Hz, 1), 7.76 (s, 1), 7.40 (d, J = 12.4 Hz, 1), 5.18 (m, 1), 3.93 (s, 3), 2.97 (t, J = 10.4 Hz, 2), 2.63 (s, 3), 2.39 (m, 2), 1.60 (s, 3), 1.54 (s, 3). *m/z* (LC-MS) 317.14 [M + H]^+^.

**6-methoxy-2-methyl-3-propionylquinoxaline 1,4-dioxide (SRI46):** was obtained as an orange solid in <5% yield. ^1^H NMR (400 MHz, CDCl_3_) δ 8.52 (d, J = 12.4 Hz, 1), 7.80 (s, 1), 7.42 (d, J =12.4 Hz, 1), 3.99 (s, 3), 2.70 (m, 4), 2.72 (s, 3), 1.29 (t, J = 10.0 Hz, 3). *m/z* (LC-MS) 317.13 [M + H]^+^.

**(E)-3-(5,9-dimethyldeca-4,8-dienoyl)-6-methoxy-2-methylquinoxaline 1,4-dioxide (SRI47):** was obtained as an orange solid in <5% yield. ^1^H NMR (400 MHz, CDCl_3_) ^1^H NMR (400 MHz, CDCl_3_) δ 8.48 (d, J = 12.4 Hz, 1), 7.80 (s, 1), 7.42 (d, J = 12.4 Hz, 1), 5.15 (m, 2), 4.00 (s, 3), 3.03 (t, J = 10 Hz, 2), 2.70 (s, 3), 2.45 (m, 2), 2.01 (m, 4), 1.60 (m, 9). *m/z* (LC-MS) 385.22 [M + H]^+^.

**(E)-3-(5,9-dimethyldeca-4,8-dienoyl)-2,6-dimethylquinoxaline 1,4-dioxide (SRI48):** was obtained as an orange solid in <5% yield. ^1^H NMR (400 MHz, CDCl_3_) δ 8.43 (m, 2), 7.67 (m 1), 5.15 (m, 2), 3.04 (t, J = 10.0 Hz, 2), 2.70 (s, 3), 2.62 (s, 3), 2.47 (m, 2), 2.01 (m, 4), 1.60 (m, 9). *m/z* (LC-MS) 369.24 [M + H]^+^.

**(E)-6-chloro-3-(5,9-dimethyldeca-4,8-dienoyl)-2-methylquinoxaline 1,4-dioxide (SRI49):** was obtained as an orange solid in <5% yield. ^1^H NMR (400 MHz, CDCl_3_) δ 8.59 (s, 1), 8.41 (d, J = 12.0 Hz, 1), 7.78 (d, J = 12.0 Hz, 1), 5.15 (m, 2), 3.05 (t, J = 10 Hz, 2), 2.70 (s, 3), 2.45 (m, 2), 2.05 (m, 4), 1.71 (m, 9). *m/z* (LC-MS) 389.20 [M + H]^+^.

**3-methyl-6-nitro-2-pentanoylquinoxaline 1,4-dioxide (SRI50):** was obtained as an orange solid in <5% yield ^1^H NMR (400 MHz, CDCl_3_) δ 9.41 (s, 1H), 8.72 (d, J = 9.2 Hz, 2), 8.60 (d, J = 9.2 Hz, 1), 2.99 (t, J = 10.0 Hz, 2), 2.54 (s, 3), 1.77 (m, 2), 1.46 (m, 2), 0.96 (t, J = 10.0 Hz, 3). *m/z* (LC-MS) 306.15 [M + H]^+^.

**(E)-2-(5,9-dimethyldeca-4,8-dienoyl)-3-methylquinoxaline 1,4-dioxide (SRI51):** was obtained as an orange solid in <5% yield ^1^H NMR (400 MHz, CDCl_3_) δ 8.61 (d, J = 9.2 Hz, 1), 8.59 (d, J = 9.2 Hz, 1), 8.79 (m, 2), 5.15 (m, 2), 3.05 (t, J = 10 Hz, 2), 2.72 (s, 3), 2.49 (m, 2), 2.05 (m, 4), 1.71 (m, 9). *m/z* (LC-MS) 355.22 [M + H]^+^.

**2-(ethoxycarbonyl)-3-methylquinoxaline 1,4-dioxide (SRI53):** was obtained as brown solid in 60% yield. ^1^H NMR (400 MHz, CDCl_3_) δ 8.42 (m, 2), 7.96 (m, 2), 4.50 (q, J = 9.2 Hz, 2), 3.29 (s, 3), 2.49 (m, 2), 2.05 (m, 4), 1.34 (t, J = 9.2 Hz, 3). *m/z* (LC-MS) 249.20 [M + H]^+^.

**2-carboxy-3-methylquinoxaline 1,4-dioxide (SRI53):** was obtained as a beige solid in 40% yield. ^1^H NMR (400 MHz, CDCl_3_) δ 8.43 (m, 2), 7.96 (m, 2), 3.29 (s, 3), 2.47 (s, 3). *m/z* (LC-MS) 221.09 [M + H]^+^.

**6,7-dichloro-2-methyl-3-pentanoylquinoxaline 1,4-dioxide (SRI54):** was obtained as an orange solid in 15% yield. ^1^H NMR (400 MHz, CDCl_3_) δ 8.73 (s, 1), 8.65 (s, 1), 2.97 (t, J = 7.6 Hz, 2), 2.49 (s, 3), 1.75 (m, 2), 1.43 (m, 2), 0.96 (t, J = 7.2 Hz, 3). *m/z* (LC-MS) 329.21 [M + H]^+^.

**6,7-dichloro-2-methyl-3-nicotinoylquinoxaline 1,4-dioxide (SRI55):** was obtained as an orange solid in 20% yield. ^1^H NMR (400 MHz, CDCl_3_) δ 8.99 (s, 1H), 8.89 (d, J = 6 Hz, 1H), 8.79 (s, 1H), 8.64 (s, 1H), 8.20 (d, J = 10.4 Hz, 1H), 7.55 (m, 1H), 2.50 (s, 3H). *m/z* (LC-MS) 350.09 [M + H]^+^.

**(E)-6,7-dichloro-2-(5,9-dimethyldeca-4,8-dienoyl)-3-methylquinoxaline 1,4-dioxide (SRI56):** was obtained as an orange solid in <5% yield. ^1^H NMR (400 MHz, CDCl_3_) δ 8.74 (s, 1H), 8.65 (s, 1H), 5.14 (m, 2H), 3.05 (t, J = 10 Hz, 2H), 2.60 (s, 3H), 2.49 (m, 2H), 2.05 (m, 4H), 1.71 (m, 9H). *m/z* (LC-MS) 423.15 [M + H]^+^.

**6,7-dichloro-2-methyl-3-(5-methylhex-4-enoyl)quinoxaline 1,4-dioxide (SRI57):** was obtained as an orange solid in 12% yield. ^1^H NMR (400 MHz, CDCl_3_) δ 8.73 (s, 1), 8.65 (s, 1), 5.18 (m, 1), 2.93 (t, J = 10.4 Hz, 2), 2.68 (s, 3), 2.42 (m, 2), 1.60 (s, 3), 1.55 (s, 3). *m/z* (LC-MS) 355.11 [M + H]^+^.

**6,7-dichloro-2-(cyclopropanecarbonyl)-3-methylquinoxaline 1,4-dioxide (SRI58):** was obtained as an orange solid in 17% yield. ^1^H NMR (400 MHz, CDCl_3_) δ 8.73 (s, 1), 8.65 (s, 1), 2.97 (t, J = 7.6 Hz, 2), 2.49 (m, 4), 1.43 (m, 2), 1.25 (m, 2). *m/z* (LC-MS) 313.11 [M + H]^+^.

**6,7-dichloro-2-hexanoyl-3-methylquinoxaline 1,4-dioxide (SRI59):** was obtained as an orange solid in 11% yield. ^1^H NMR (400 MHz, CDCl_3_) δ 8.73 (s, 1), 8.65 (s, 1), 2.96 (t, J = 10.4 Hz, 2), 2.49 (s, 3), 1.78 (m, 2), 1.37 (m, 4), 0.91 (t, J = 8.4 Hz, 3). *m/z* (LC-MS) 343.13 [M + H]^+^.

**6,7-dichloro-2-methyl-3-(3-methylbutanoyl)quinoxaline 1,4-dioxide (SRI60):** was obtained as an orange solid in 18% yield. ^1^H NMR (400 MHz, CDCl_3_) δ 8.73 (s, 1), 8.66 (s, 1), 2.89 (d, J = 8.8 Hz, 2), 2.50 (s, 3), 2.39 (m, 1), 1.05 (d, J = 8.8 Hz, 6H). *m/z* (LC-MS) 329.12 [M + H]^+^.

**6,7-dichloro-2-methyl-3-(pent-4-enoyl)quinoxaline 1,4-dioxide (SRI61):** was obtained as an orange solid in 12% yield. ^1^H NMR (400 MHz, CDCl_3_) δ 8.73 (s, 1), 8.66 (s, 1), 5.02 (m, 2), 3.03 (m, 2), 2.71 (s, 3), 2.49 (m, 2). *m/z* (LC-MS) 327.07 [M + H]^+^.

**2-benzoyl-6,7-dichloro-3-methylquinoxaline 1,4-dioxide (SRI62):** was obtained as an orange solid in 21% yield. ^1^H NMR (400 MHz, CDCl_3_) δ 8.79 (s, 1), 8.66 (s, 1), 7.87 (d, J = 7.6 Hz, 2), 7.70 (t, J = 7.6 Hz, 1), 7.54 (d, J = 7.6 Hz, 2), 2.48 (s, 3), 2.49 (m, 2). *m/z* (LC-MS) 349.09[M + H]^+^.
