# Supplementary material for: Combining Metabolite-Based Pharmacophores with Bayesian Machine Learning Models for Mycobacterium tuberculosis Drug Discovery
Source: PLoS One. 2015 Oct 30;10(10):e0141076. doi: 10.1371/journal.pone.0141076 (PMC4627656; doi:10.1371/journal.pone.0141076)
Supplement: S1 Fig — (PDF) [file pone.0141076.s002.pdf]

PLOS ONE

Supplemental data

Combining Metabolite-Based Pharmacophores with Bayesian Machine Learning Models for *Mycobacterium tuberculosis* Drug Discovery

Sean Ekins[1,2*], Peter B. Madrid[3*], Malabika Sarker[3], Shao-Gang Li[4], Nisha Mittal[4], Xin Wang[4], Thomas P. Stratton[4], Matthew Zimmerman,[5] Carolyn Talcott[3], Pauline Bourbon[3], Mike Travers[1], Maneesh Yadav[3] and Joel S. Freundlich[4*]

[1]Collaborative Drug Discovery Inc., 1633 Bayshore Highway, Suite 342, Burlingame, CA 94010, USA.

[2]Collaborations in Chemistry, 5616 Hilltop Needmore Road, Fuquay-Varina, NC 27526, USA.

[3]SRI International, 333 Ravenswood Avenue, Menlo Park, CA 94025, USA.

[4]Departments of Pharmacology & Physiology and Medicine, Center for Emerging and Reemerging Pathogens, Rutgers University – New Jersey Medical School, 185 South Orange Avenue, Newark, NJ 07103, USA.

[5]Public Health Research Institute, Rutgers University – New Jersey Medical School, Newark, NJ 07103, USA.

*Authors contributed equally; Addresses for correspondence: Sean Ekins, Collaborative Drug Discovery, 1633 Bayshore Highway, Suite 342, Burlingame, CA 94010, USA. E-

mail:ekinssean@yahoo.com, Phone: 215-687-1320; Peter Madrid, SRI International, 333 Ravenswood Avenue, Menlo Park, CA 94025, USA. E-mail: peter.madrid@sri.com; Joel S. Freundlich, Departments of Pharmacology & Physiology and Medicine, Center for Emerging and Reemerging Pathogens, Rutgers University – New Jersey Medical School, 185 South Orange Avenue Newark, NJ 07103, USA. E-mail: freundjs@rutgers.edu, Phone: 973-972-7165.

Figure S1. Pharmacophores used for database searches.

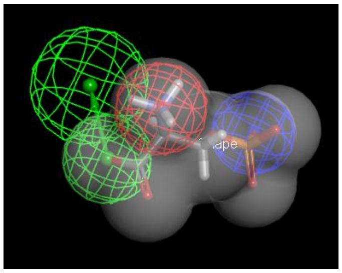

3-phosphoserine

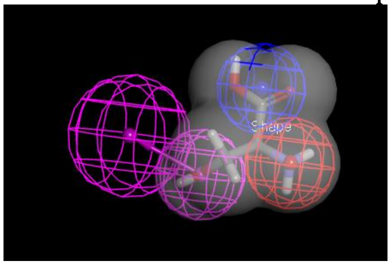

L-serine

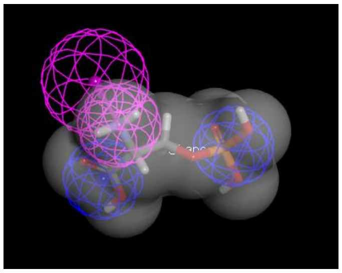

L-(or D-)-O-phosphoserine

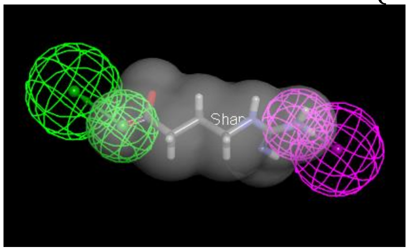

4-guanidinobutyrate

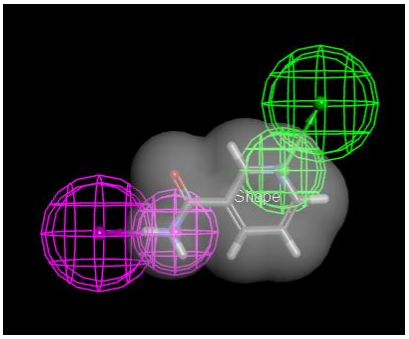

nicotinamide

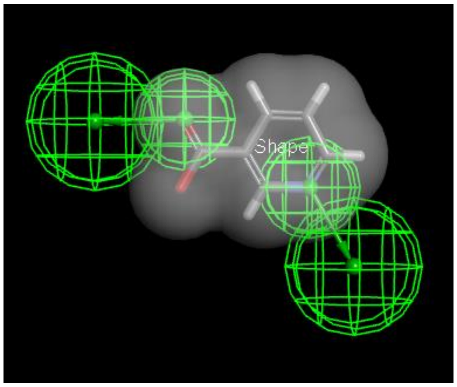

nicotinate

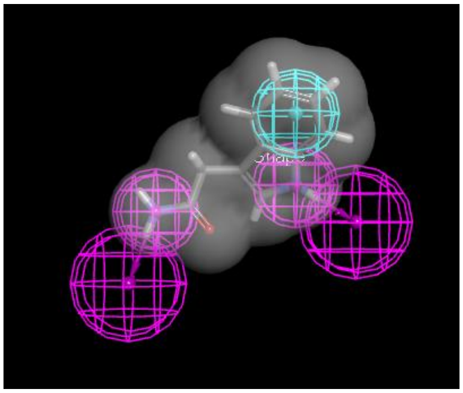
indole-3-acetamide

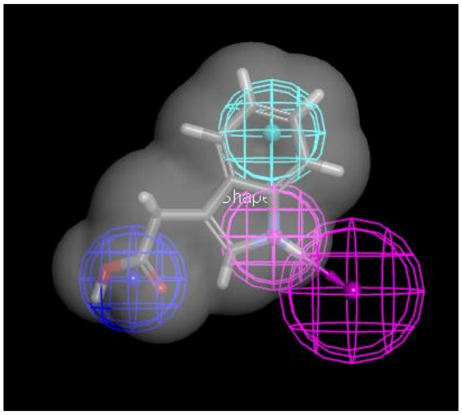
indole-3-acetate

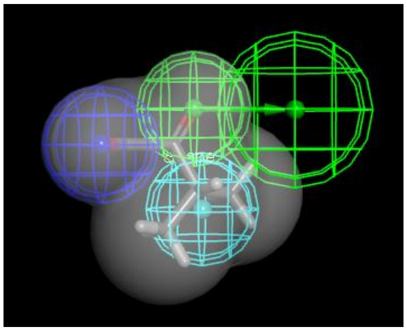
2-oxoisovalerate

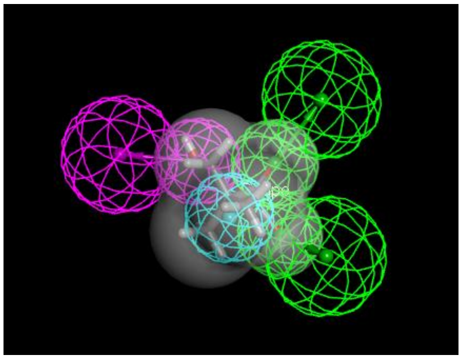
2-dehydropantoate

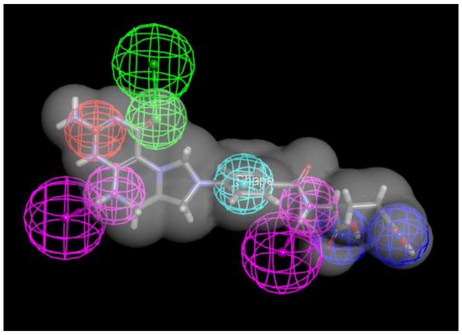
5,10-methylenetetrahydrofolate

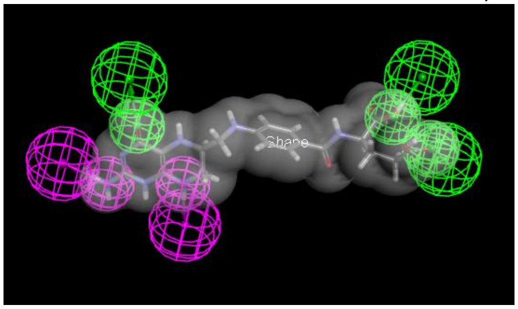

tetrahydrofolate

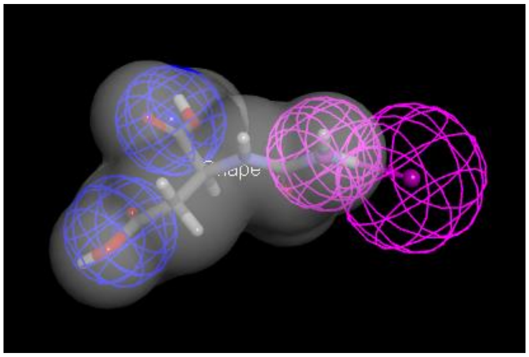
N-carbamoyl-L-aspartate

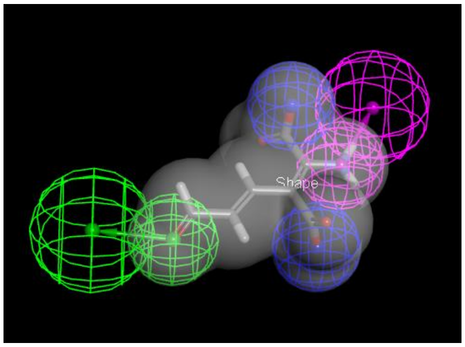

aminocarboxymuconate semialdehyde

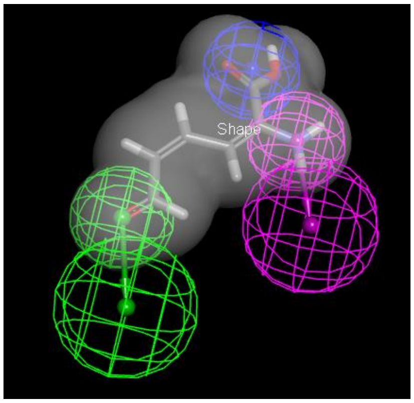

2-aminomuconate semialdehyde

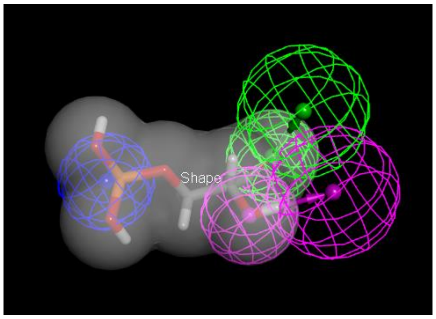

D-glyceraldehyde-3-phosphate

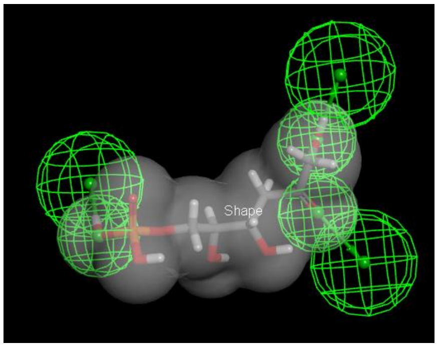

D-fructose-6-phosphate

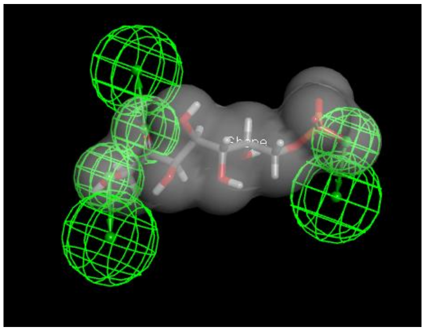
D-sedoheptulose-7-phosphate

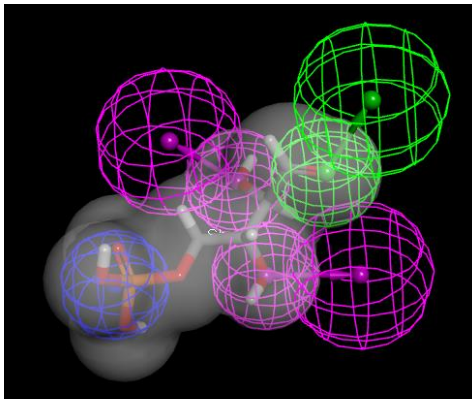
D-erythrose-4-phosphate

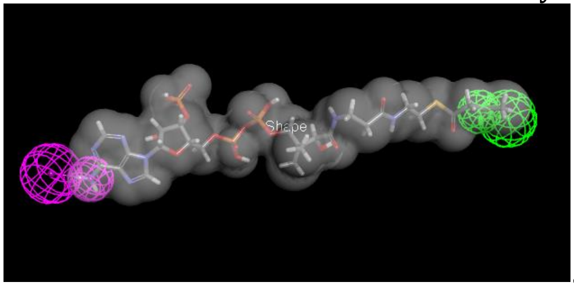

acetoacetyl-CoA

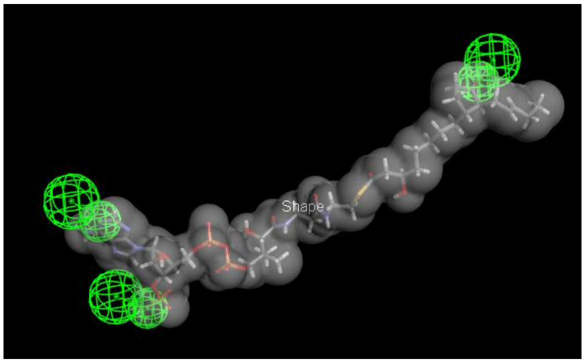

OPC8-3-hydroxyacyl-CoA

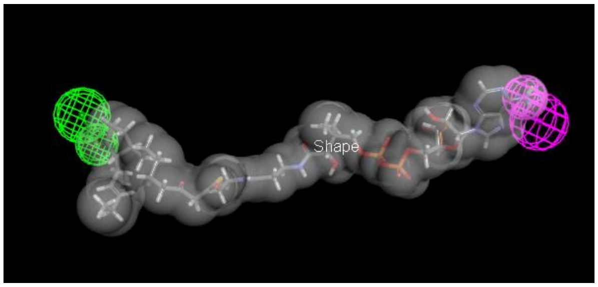

OPC8-3-ketoacyl-CoA

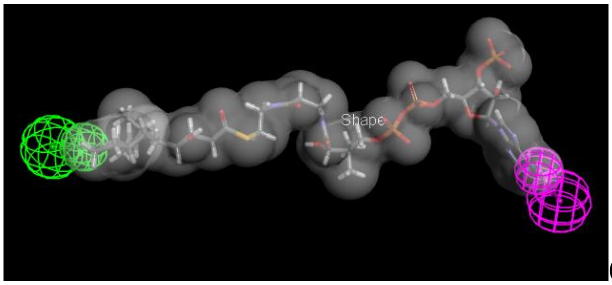

OPC6-3-hydroxylacyl-CoA

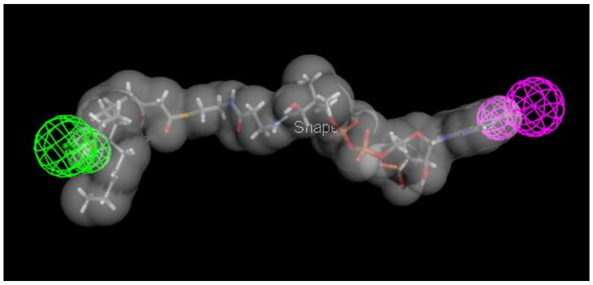

OPC6-3-ketoacyl-CoA

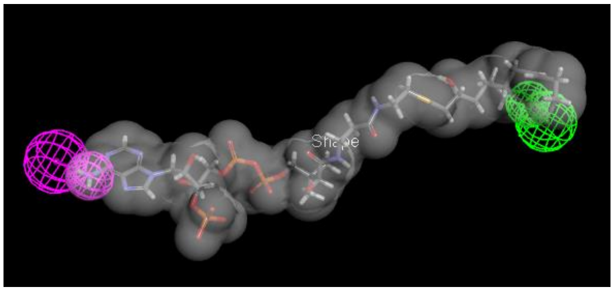

OPC4-3-hydroxylacyl-CoA

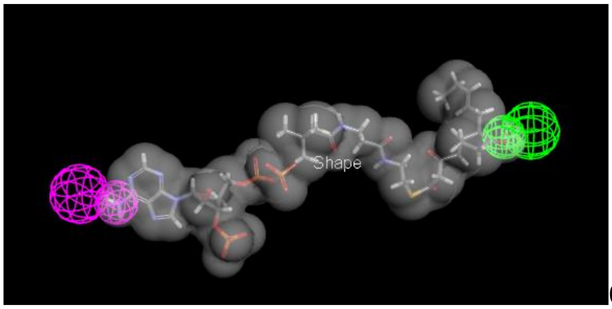

OPC4-3-ketoacyl-CoA

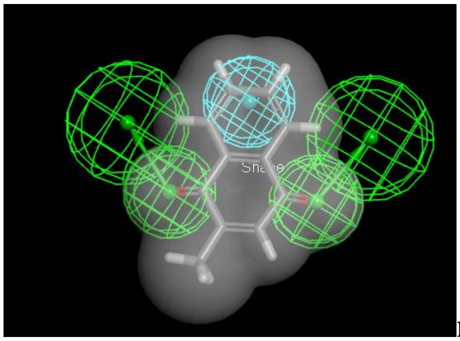
menadione

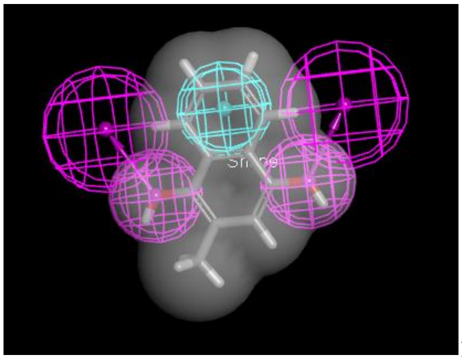
menadiol

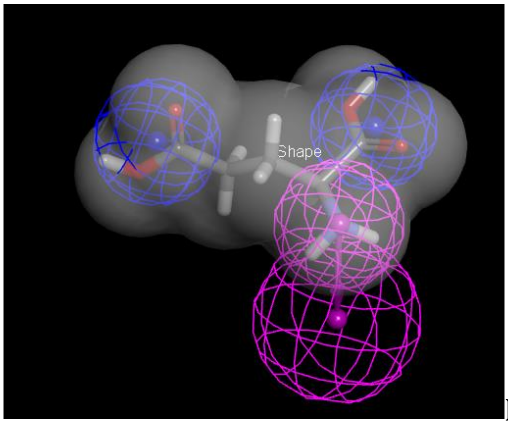

L-glutamate γ-semialdehyde

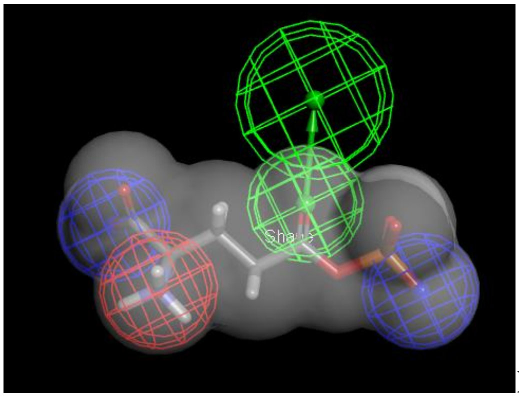
L-glutamate-5-phosphate

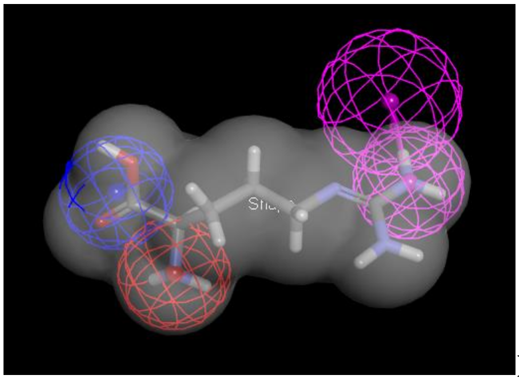
L-arginine

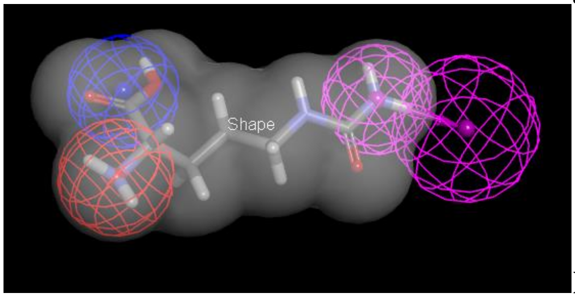

L-citrulline

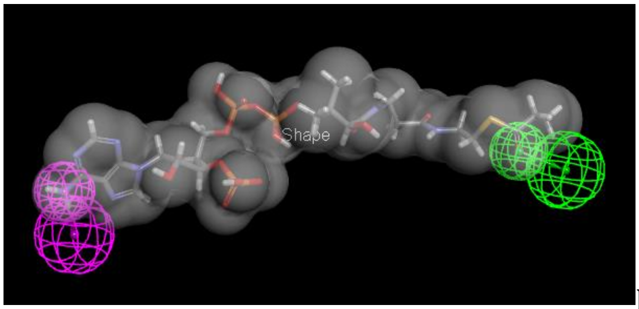

propanoyl-CoA

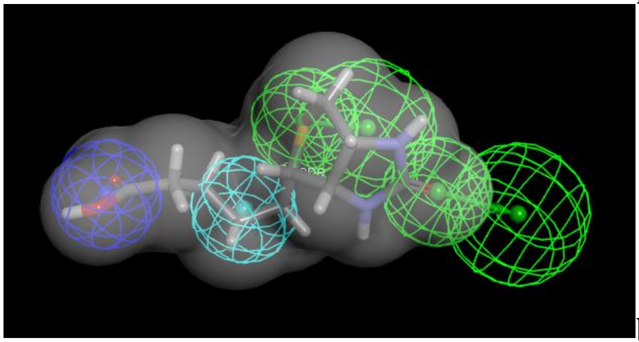

biotin

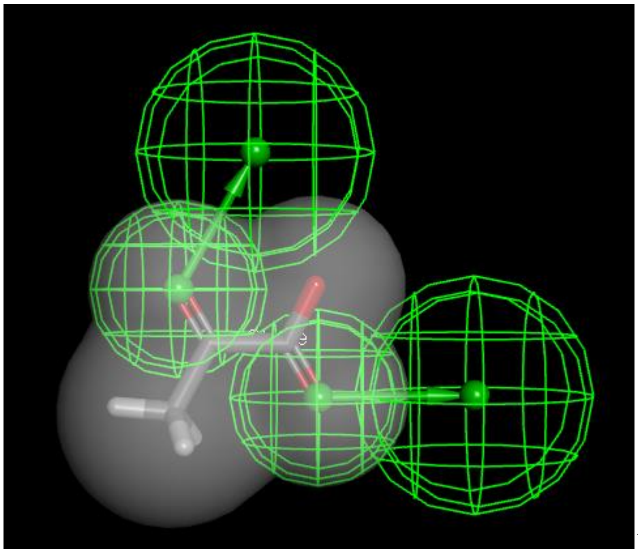

pyruvate

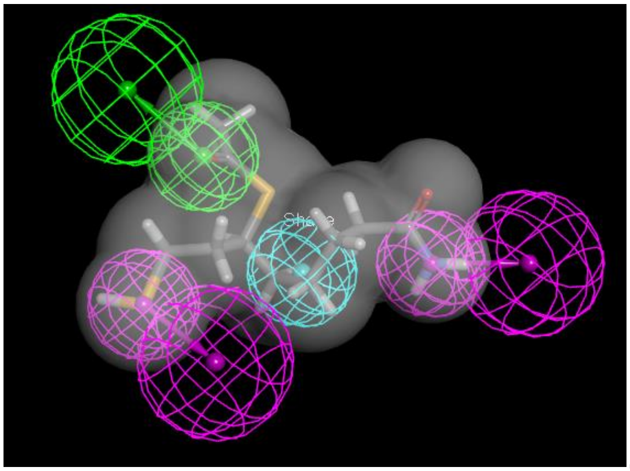

S-acetyldihydrolipoamide

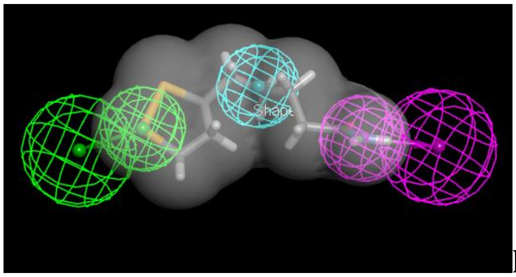
lipoamide

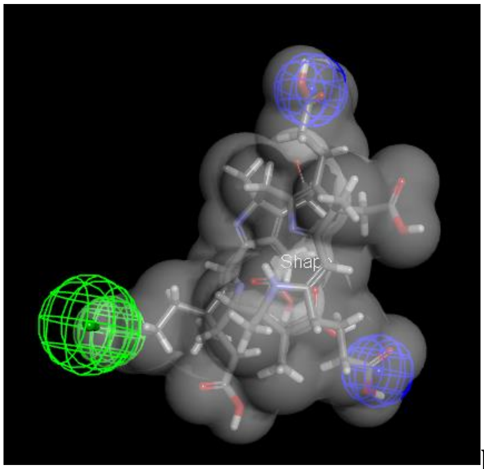
precorrin-6B

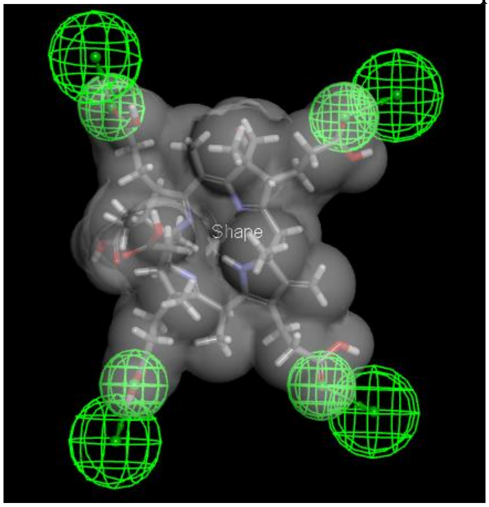

precorrin-8x

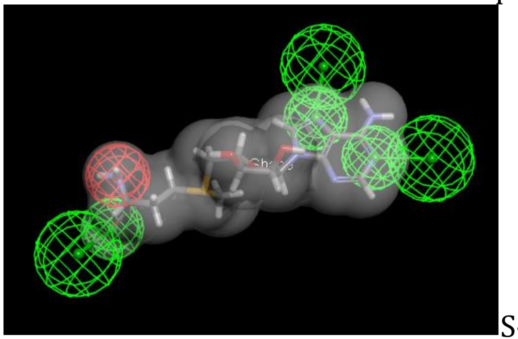
S-adenosyl-L-methionine

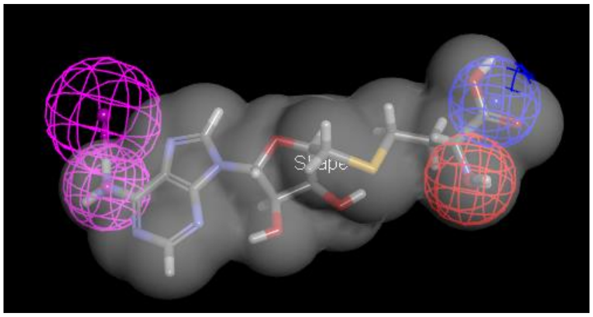
S-adenosyl-L-homocysteine

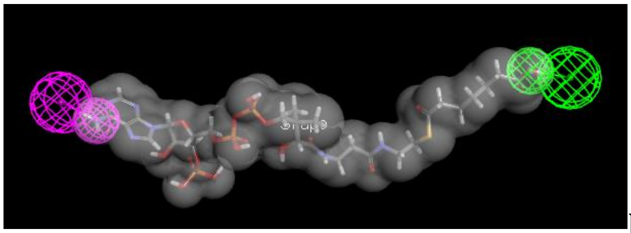
pimeloyl-CoA

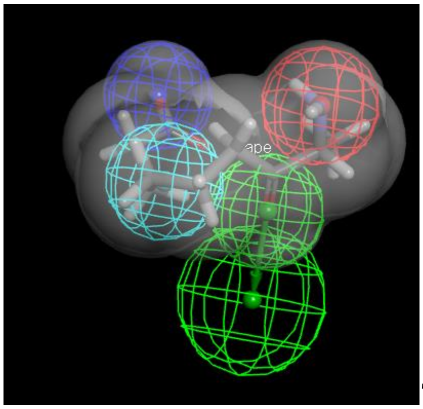
7-keto-8-aminopelargonate

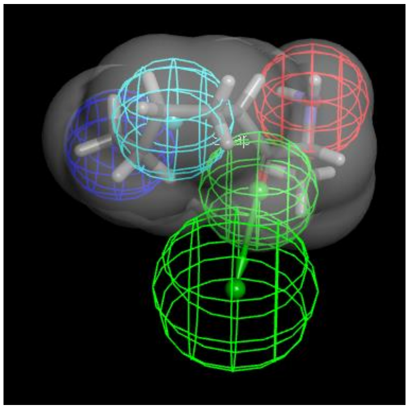
7-keto-8-aminopelargonate

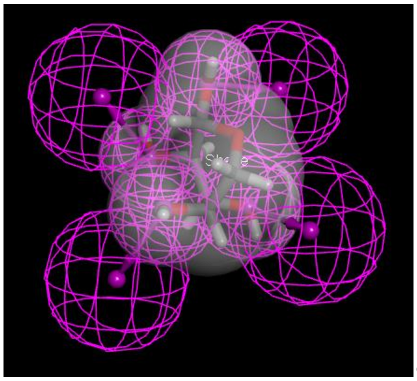
α-D-xylopyranose

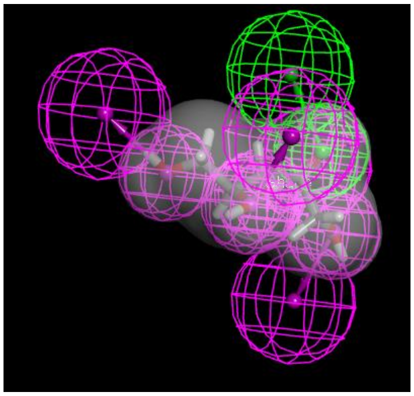
D-xylulose

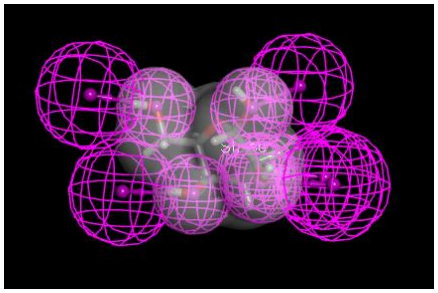
β-D-glucose

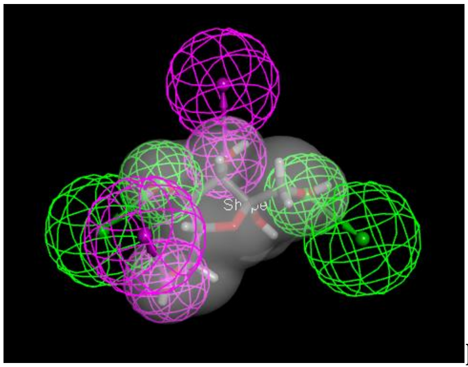
D-fructose

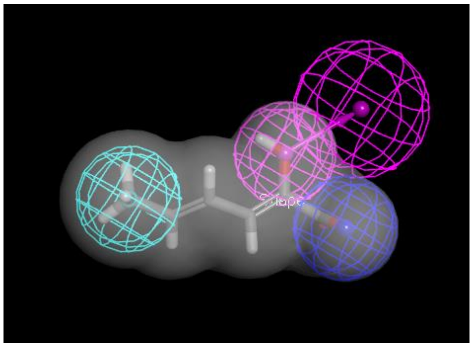
2-hydroxyhexa-2,4,-dienoate

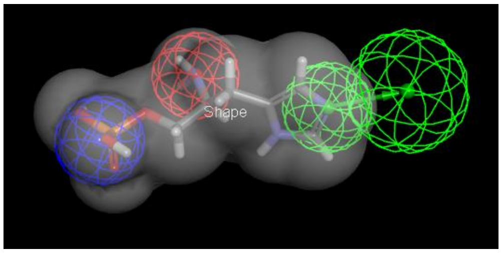
L-histidinol-phosphate

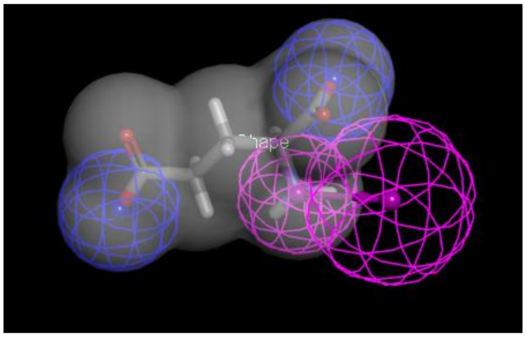
L-glutamate

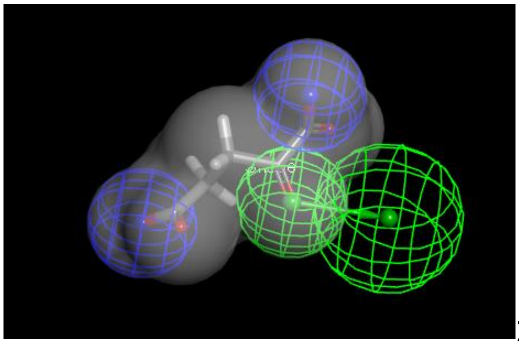
2-oxoglutarate

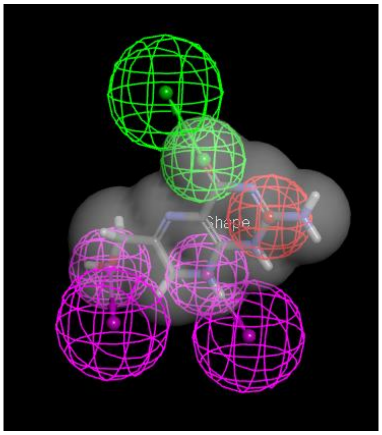

6-hydroxymethyl-7,8-dihydropterin

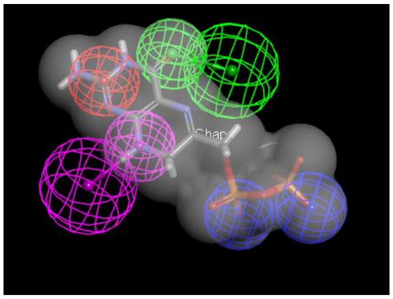
6-hydroxymethyl-dihydropterin diphosphate

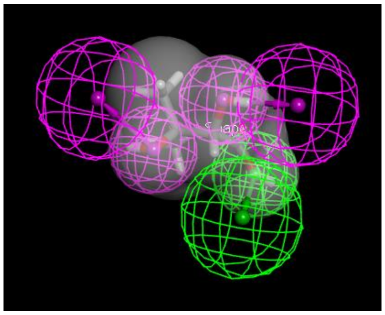

2,3-dihydroxy-3-methylbutanoate

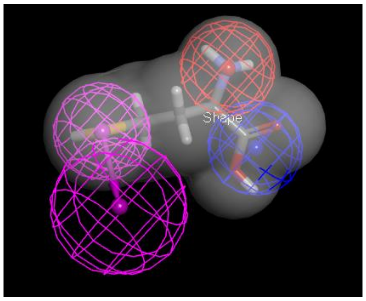

L-homocysteine

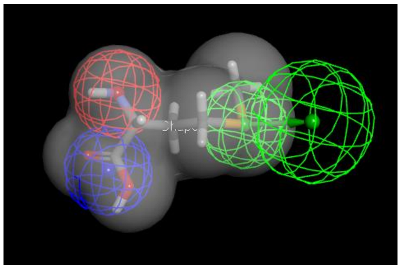
L-methionine

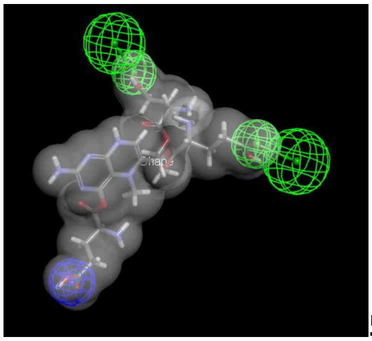
5-methyltetrahydropteroyltri-L-glutamate

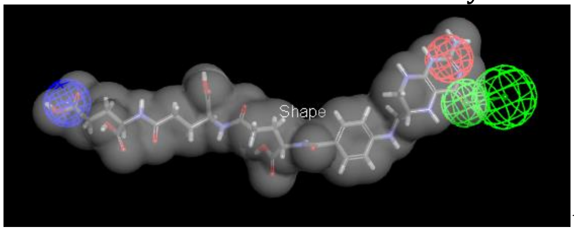
tetrahydropteroyl tri-L-glutamate

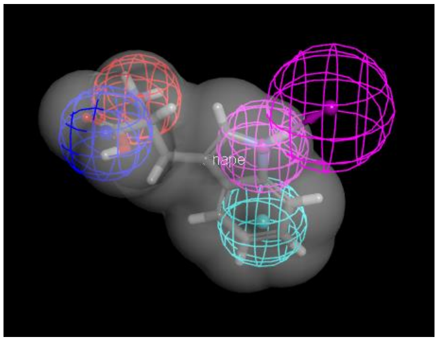
L-tryptophan

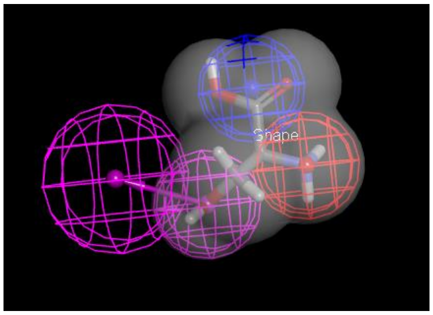
D-serine

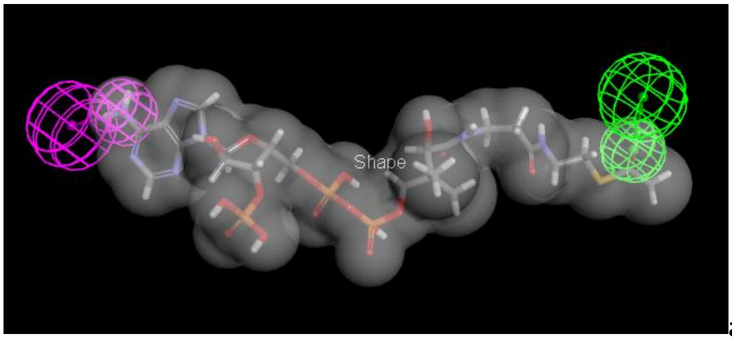
acetyl-CoA

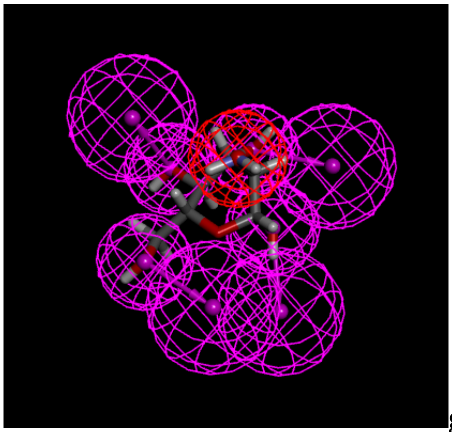
glucosamine

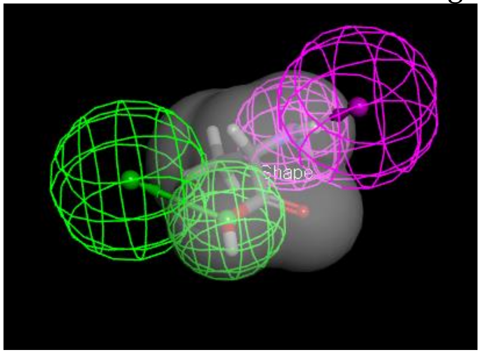
L-alanine

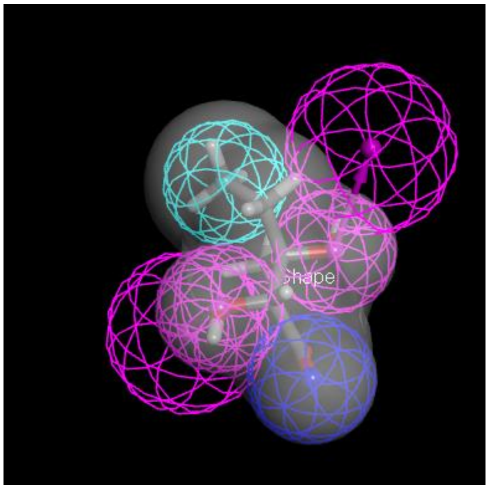
2,3-dihydroxy-3-methylvalerate

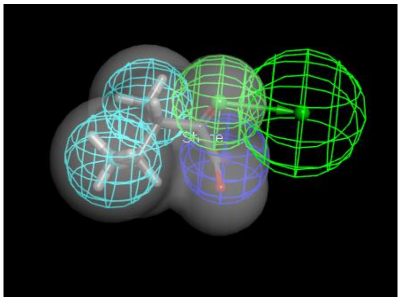
2-keto-3-methyl-valerate

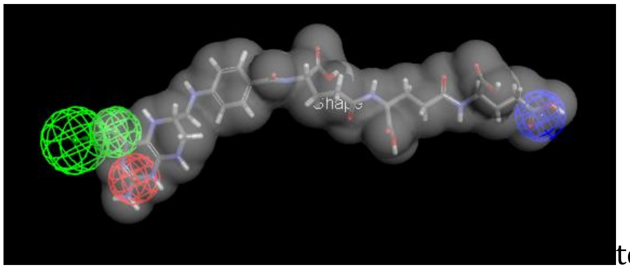
tetrahydropteroyl tri-L-glutamate
