# Supplementary material for: Combining Metabolite-Based Pharmacophores with Bayesian Machine Learning Models for Mycobacterium tuberculosis Drug Discovery
Source: PLoS One. 2015 Oct 30;10(10):e0141076. doi: 10.1371/journal.pone.0141076 (PMC4627656; doi:10.1371/journal.pone.0141076)

PLOS ONE

Supplemental data

Combining Metabolite-Based Pharmacophores with Bayesian Machine Learning Models for *Mycobacterium tuberculosis* Drug Discovery

Sean Ekins[1,2*], Peter B. Madrid[3*], Malabika Sarker[3], Shao-Gang Li[4], Nisha Mittal[4], Xin Wang[4], Thomas P. Stratton[4], Matthew Zimmerman,[5] Carolyn Talcott[3], Pauline Bourbon[3], Mike Travers[1], Maneesh Yadav[3] and Joel S. Freundlich[4*]

[1]Collaborative Drug Discovery Inc., 1633 Bayshore Highway, Suite 342, Burlingame, CA 94010, USA.

[2]Collaborations in Chemistry, 5616 Hilltop Needmore Road, Fuquay-Varina, NC 27526, USA.

[3]SRI International, 333 Ravenswood Avenue, Menlo Park, CA 94025, USA.

[4]Departments of Pharmacology & Physiology and Medicine, Center for Emerging and Reemerging Pathogens, Rutgers University – New Jersey Medical School, 185 South Orange Avenue, Newark, NJ 07103, USA.

[5]Public Health Research Institute, Rutgers University – New Jersey Medical School, Newark, NJ 07103, USA.

*Authors contributed equally; Addresses for correspondence: Sean Ekins, Collaborative Drug Discovery, 1633 Bayshore Highway, Suite 342, Burlingame, CA 94010, USA. E-

mail:ekinssean@yahoo.com, Phone: 215-687-1320; Peter Madrid, SRI International, 333 Ravenswood Avenue, Menlo Park, CA 94025, USA. E-mail: peter.madrid@sri.com; Joel S. Freundlich, Departments of Pharmacology & Physiology and Medicine, Center for Emerging and Reemerging Pathogens, Rutgers University – New Jersey Medical School, 185 South Orange Avenue Newark, NJ 07103, USA. E-mail: freundjs@rutgers.edu, Phone: 973-972-7165.

Figure S2. PCA with the TB mobile dataset showing the 3 hits in yellow – 88.8% of variance explained in 3 PCs.

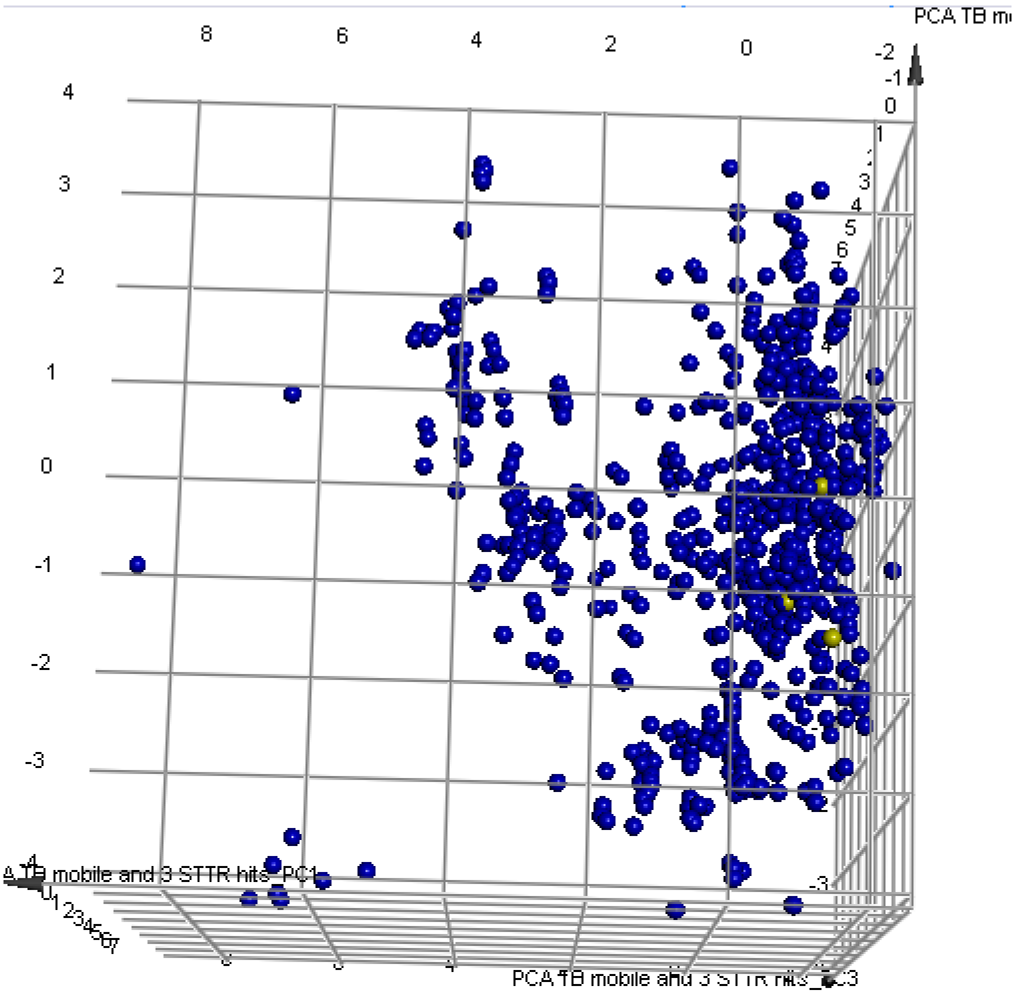

Supplement: S2 Fig — (PDF) [file pone.0141076.s003.pdf]
